# Recombinant Lipases and Phospholipases and Their Use as Biocatalysts for Industrial Applications

**DOI:** 10.3390/ijms160920774

**Published:** 2015-09-01

**Authors:** Grazia M. Borrelli, Daniela Trono

**Affiliations:** Consiglio per la Ricerca in Agricoltura e l’Analisi dell’Economia Agraria, Centro di Ricerca per la Cerealicoltura, S.S. 673 Km 25, 200-71122 Foggia, Italy; E-Mail: graziamaria.borrelli@entecra.it

**Keywords:** lipases, phospholipases, biocatalysis, heterologous expression, protein engineering, industrial applications

## Abstract

Lipases and phospholipases are interfacial enzymes that hydrolyze hydrophobic ester linkages of triacylglycerols and phospholipids, respectively. In addition to their role as esterases, these enzymes catalyze a plethora of other reactions; indeed, lipases also catalyze esterification, transesterification and interesterification reactions, and phospholipases also show acyltransferase, transacylase and transphosphatidylation activities. Thus, lipases and phospholipases represent versatile biocatalysts that are widely used in various industrial applications, such as for biodiesels, food, nutraceuticals, oil degumming and detergents; minor applications also include bioremediation, agriculture, cosmetics, leather and paper industries. These enzymes are ubiquitous in most living organisms, across animals, plants, yeasts, fungi and bacteria. For their greater availability and their ease of production, microbial lipases and phospholipases are preferred to those derived from animals and plants. Nevertheless, traditional purification strategies from microbe cultures have a number of disadvantages, which include non-reproducibility and low yields. Moreover, native microbial enzymes are not always suitable for biocatalytic processes. The development of molecular techniques for the production of recombinant heterologous proteins in a host system has overcome these constraints, as this allows high-level protein expression and production of new redesigned enzymes with improved catalytic properties. These can meet the requirements of specific industrial process better than the native enzymes. The purpose of this review is to give an overview of the structural and functional features of lipases and phospholipases, to describe the recent advances in optimization of the production of recombinant lipases and phospholipases, and to summarize the information available relating to their major applications in industrial processes.

## 1. Introduction

Biocatalysis is defined as the use of enzymes to catalyze biochemical reactions. Enzymes are complex proteins that are produced by living organisms to bring about the specific biochemical reactions that are necessary to develop and maintain life. Although enzymes are produced by living cells, they can also be extracted from organisms and used for industrial, pharmaceutical and biotechnological applications [1,2,3]. Compared to classical chemical catalysts, the use of enzymes offers various advantages, as they: (i) specifically direct the reaction course toward a predetermined product, thus reducing the risk of side reactions; (ii) work under mild conditions of temperature and pH; (iii) avoid the production of toxic end-products. Moreover, if immobilized on various matrices and supports, enzymes can be reused several times, so this reduces the cost and improves the performance of the biocatalyst [4]. Overall, the use of enzymes on industrial scales guarantees energy saving and avoids pollution, thus holding promise for green and economically sustainable alternative strategies in industrial transformations.

Commercially available enzymes are derived from animals, plants and microorganisms. The greater part of industrial enzymes (more than 50%) is, however, of microbial origin [2], as, compared to enzymes derived from animal and plant sources, they are more stable and cover a greater variety of catalytic activities. Moreover, microorganisms can rapidly grow in inexpensive media, thus reaching high yields, and representing an always available source, as their growth is not affected by seasonal fluctuations. Nature provides a vast biodiversity of microbial resources. Microorganisms have great adaptative capabilities, even in inhospitable environments, such as in the waters of the deep-seas, the Dead Sea, the Antarctic, the alkaline lakes, and in hydrothermal and volcanic vents, and contaminated soils, which provide impressive potential for enzymes with specific characteristics [5]. These properties have been exploited by traditional culture-based methods and by metagenomics, which is the culture-independent genomic analysis of microbial communities. This has allowed new biocatalysts to be identified that better fulfill the technical specifications of industrial processes.

The majority (more than 75%) of enzymes currently used in industrial processes are hydrolytic in action [6]. Among these, the lipases and phospholipases are the enzymes that are used for lipid modifications. Lipases represent the third most commercialized enzymes, after proteases and carbohydrases [7], and their production has constantly increased, such that they now account for more than one-fifth of the global enzyme market. Lipases form an integral part of the industries ranging from biodiesels, food, nutraceuticals and detergents with minor applications also in bioremediation, agriculture, cosmetics, leather and paper industries [8]. For the phospholipases, several genes have been cloned, expressed and patented in the literature, although their major applications have only been in a few industries, such as food, nutraceuticals and oil degumming [9].

The great biotechnological potential of lipases and phospholipases is because they have versatile applications by virtue of their unique properties. Indeed, both lipases and phospholipases catalyze the hydrolysis of ester bonds in the presence of interfaces, and under nonaqueous conditions they can catalyze various reverse reactions. Moreover, these biocatalysts have broad substrate specificity, enantio-selectivity, as well as stability in organic solvents and at extreme temperatures and pHs. Since the 1980s, various lipases and phospholipases have been produced commercially. Porcine and human pancreas were the first sources of lipases and phospholipases used in food processing, but nowadays, the main sources of the commercially available enzymes are yeasts, fungi and bacteria.

However, despite the great potential of lipases and phospholipases, their use in industry is sometimes limited, as the traditional purification strategies from microbe cultures may have some disadvantages, which include non-reproducibility and low yields. Lipases and phospholipases from natural sources also do not always meet the requirements for industrial biocatalysis in terms of their activities, specificities and stabilities. As a consequence, there is great interest in methodologies to develop the reproducible and high-yield production of competitive biocatalysts for industrial applications, with the recent introduction and development of new DNA recombinant technologies and the advances in genetic engineering proving to be very helpful to address these challenges. For instance, heterologous expression systems are increasingly being used to overproduce recombinant lipases and phospholipases, thus overcoming the constraints related to the production of native enzymes [10]. Moreover, the application of protein engineering has enabled scientists to rapidly tailor the properties of biocatalysts, thus generating new lipase and phospholipase variants that have catalytic and stability properties that better fit the needs of industrial processes [11,12,13].

Thus, in the present review we first take a broader view of the structural and catalytic features of both lipases and phospholipases, as well as of their roles in biocatalysis. We then focus on such topics as heterologous systems for high-level production of recombinant lipases and phospholipases, and protein engineering strategies for the improvement of their catalytic and stability properties. Finally, an overview of the applications of recombinant lipases and phospholipases in the most relevant industrial processes is given. Although a broad subject such as this cannot be covered completely in this review, the following sections will explore each of these concepts in greater detail.

## 2. General Overview of Lipases and Phospholipases

### 2.1. Lipases

#### 2.1.1. Reactions Catalyzed by Lipases

**Figure 1:**
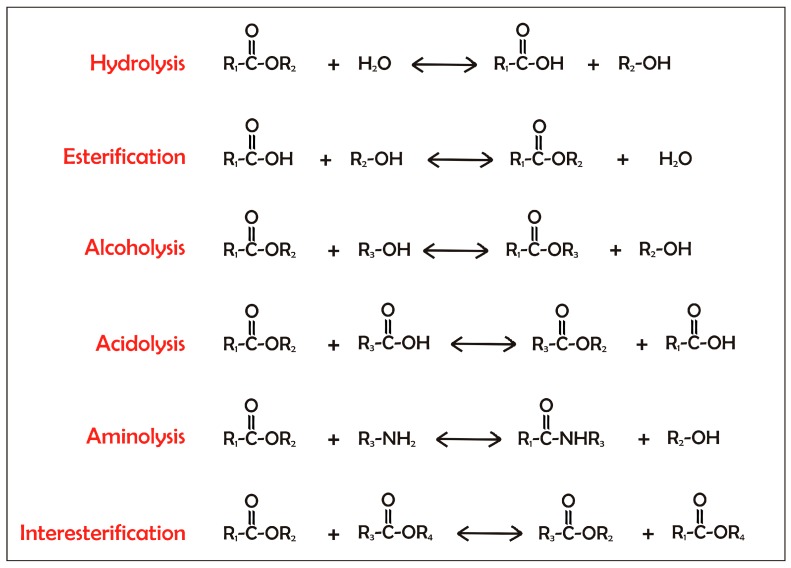
Reactions catalyzed by lipases. Lipases catalyze the cleavage of carboxyl ester bonds. This reaction occurs in the presence of water as substrate or product in hydrolysis and esterification, respectively, of an alcohol in alcoholysis, of an organic acid in acidolysis, of ammonia in aminolysis, and between two different esters in interesterification.

Under natural conditions, such as in the presence of excess water, lipases act at the organic–aqueous interface to catalyze the hydrolysis of carboxylate ester bonds and the release free fatty acids (FFAs) and organic alcohols (Figure 1). As the equilibrium between the forward and reverse reactions is controlled by the water activity (*a*_w_) of the reaction mixture, under limiting water conditions the reverse reaction, the esterification, can occur (Figure 1). Under low *a*_w_, different transesterification reactions can also be achieved. The term transesterification refers to the exchange of groups between an ester and an alcohol (alcoholysis), between an ester and an acid (acidolysis), between an ester and an amine (aminolysis), or between two esters (interesterification) [14] (Figure 1).

Lipases have different kinds of selectivities toward their substrates [15]. These include: (i) Chemo-specificity. Lipases show both fatty acid (FA) specificity and lipid-class specificity. Their FA specificity is related to the release of FFAs with length or degree of unsaturation that falls within well-defined ranges. For lipid-class specificity, lipases catalyze not only hydrolysis of triacylglycerol (TAG) but also of diacylglycerol (DAG) and monoacylglycerol (MAG); (ii) Regio-specificity. Lipases can be divided into the nonspecific lipases that catalyze the complete hydrolysis of TAGs into glycerol and FFAs in a random way, thus generating DAGs and MAGs as intermediates, and the specific 1,3-lipases that hydrolyze TAGs only at the *sn*-1 and *sn*-3 positions, thus generating FFAs, 1,2-DAGs or 2,3-DAGs, and 2-MAGs. Here, 2,3-DAGs and 2-MAGs are chemically unstable, and they undergo acyl migration to give 1,3-DAGs and 1-MAGs or 3-MAGs, respectively; (iii) Enantio-selectivity. There are lipases that are able to distinguish between enantiomers in a racemic mixture. This selectivity varies with respect to the substrate, as it is connected to the nature of the ester.

#### 2.1.2. Classification of the Lipases

As shown in Table 1, the mammalian lipases comprise hepatic lipase, endothelial lipase, lipoprotein lipase, pancreatic lipase and pancreatic lipase-related protein 1–3; these all belong to the “pancreatic lipase gene family” [16,17,18]. This pancreatic lipase gene family also includes three PLA_1_s that specifically hydrolyze phospholipids [16] (see Section 2.2.2 and Table 3). Other mammalian lipases not included in this pancreatic lipase gene family are the hormone-sensitive lipase [19] and the bile salt-stimulated lipase [20] (Table 1).

**Table 1:** Classification and properties of mammalian lipases.

Type	Lipase	Acronymous	Substrate	Tissue	Function	Refs.
Pancreatic lipase gene family	Hepatic lipase	HL, HTGL, LIPC	TAG, PC	Hepatocytes	Metabolism of lipoproteins	[16]
	Endothelial lipase	LIPG, EL, EDL	TAG, PC	Endothelial cells	Metabolism of lipoproteins	[16]
	Lipoprotein lipase	LPL	TAG	Adipose tissue, other tissues	Metabolism of lipoproteins	[16]
	Pancreatic lipase	PL	TAG	Pancreatic acinar cells	Intestinal absorption of FAs	[16]
	Pancreatic lipase-related protein 1	PLRP1	No activity	Pancreatic acinar cells	Unknown	[17]
	Pancreatic lipase-related protein 2	PLRP2	TAG, PC, galactolipid	Pancreatic acinar cells	Intestinal absorption of FAs	[16]
	Pancreatic lipase-related protein 3	PLRP3	Unknown	Hepatocytes	Hepatocellular carcinoma	[18]
Other lipases	Hormone-sensitive lipase	HSL	TAG, DAG, MAG, cholesteryl esters	Adipose tissue, steroidogenic tissues	Mobilization of FAs, spermatogenesis, steroidogenesis	[19]
	Bile salt-stimulated lipase	BSSL	TAG, DAG, MAG, cholesteryl esters	Pancreatic acinar cells, breast milk	Absorption and digestion of fats	[20]

In plants, true lipases are abundant in the oil bodies of oleaginous seeds, and have also been found in cereal and bean seeds [15,21,22]. In seeds, lipolytic enzymes are needed for the mobilization of the stored FAs that need to be released to provide the energy and carbon for seedling growth [23]. For this reason, lipase activity is generally absent in ungerminated seeds, but rapidly appears after germination [22]. A putative lipase has also been identified in the plastoglobuli of chloroplasts, where it is probably involved in mobilization of FAs [24]. The milky sap produced by rubber trees, which is known as latex, also contains lipase activity [25].

In bacteria, the lipases are in Family I of the eight families of bacterial lipolytic enzymes, from I to VIII, according to the classification of Arpigny and Jaeger [26], where Families II to VIII comprise the esterases. This classification has been revised several times, and currently there are XVI families of lipolytic enzymes that are a part of the ESTHER database (http://bioweb.ensam.inra.fr/esther) [27]. As shown in Table 2, the lipases in Family I are divided further into seven subfamilies (I.1–I.7) [26,28], on the basis of (i) structural features (e.g., residues forming the catalytic site or the presence of disulphide bonds); (ii) types of secretion mechanism and requirement for lipase-specific foldases; and (iii) relationship to other enzyme families.

**Table 2:** Classification of bacterial lipases.

Subfamily	Species	Molecular Weight (kDa)	Properties	Refs.
I.1	*Acinetobacter calcoaceticus*, *Proteus vulgaris*, *Pseudomonas aeruginosa* (LipA, LipC), *Pseudomonas fragi*, *Pseudomonas wisconsinensis*, *Psudomonas fluorescens C9*, *Vibrio colera*	30–32	Requirement of “Lif” for folding, two Asp residues in the Ca^2+^-binding loop, two Cys residues forming a disulphide bridge	[26,27,28]
I.2	*Burkholderia cepacia*, *Burkholderia glumae*, *Chromobacterium viscosum*, *Pseudomonas luteola*	33	Requirement of “Lif” for folding, two Asp residues in the Ca^2+^-binding loop, two Cys residues forming a disulphide bridge	[26,27,28]
I.3	*Pseudomonas fluorescens* SIKW1	50	Absence of an N-terminal signal peptide, secretion via type I pathway	[26,27,28]
	*Serratia marcescens*	65		
I.4	*Bacillus subtilis* (LipA, LipB), *Bacillus pumilus*, *Bacillus licheniformis*	~20	AxSxG pentapeptide, absence of “lid”	[26,27,28]
I.5	*Geobacillus stearothermophilus* L1, *Geobacillus stearothermophilus* P1, *Geobacillus thermocatenulatus*, *Geobacillus thermoleovorans*	~45	Maximal activity at high pH (9.0) and temperature (65 °C)	[26,27,28]
I.6	*Staphylococcus aureus*, *Staphylococcus haemolyticus*, *Staphylococcus epidermidis*, *Staphylococcus hyicus*, *Staphylococcus xylosus*, *Staphylococcus warneri*	~75	Secretion as precursor and cleavage by extracellular protease	[26,27,28]
I.7	*Propionibacterium acnes*	33	50% similarity with the lipases from *Bacillus subtilis* and subfamily I.2	[26,27,28]
	*Streptomyces cinnamoneus*	50		

**Figure 2:**
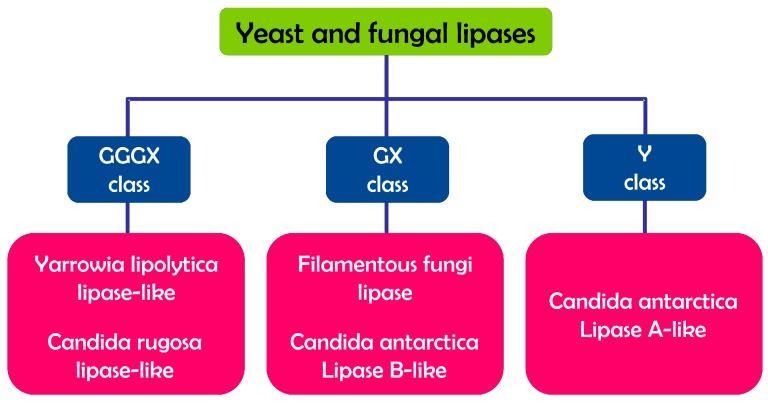
Classification of yeast and fungal lipases.

In addition to the classification of Arpigny and Jaeger [26], a new classification was more recently reported in the Lipase Engineering Database (LED) (http://www.led.uni-stuttgart.de) [29], which today includes not only bacterial, but also yeast, fungal and mammalian lipases. This classification distributes the lipases into three classes on the basis of the oxyanion hole: GX, GGGX, and Y (see Section 2.1.3) [30]. On the basis of this classification and of the amino-acid sequence similarities, yeasts and fungal lipases have been grouped into five different subclasses, two in the GX class, two in the GGGX class and one in the Y class (Figure 2) [31].

#### 2.1.3. Structural Features and Catalytic Mechanisms of Lipases

**Figure 3:**
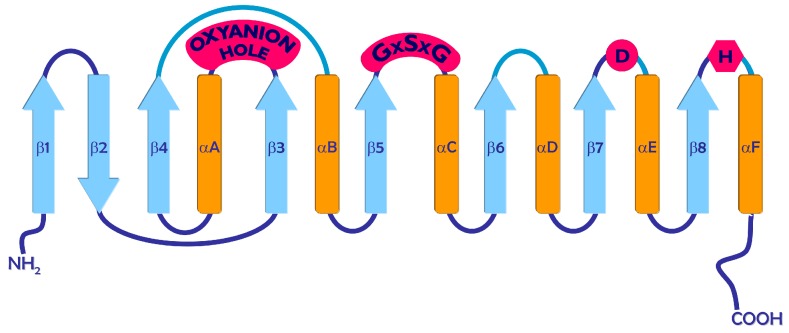
Canonical fold of α/β hydrolases. Strands are indicated by arrows and helices by cylinders. The positions of the histidine (H) and aspartate (D) residues, ad well as the GXSXG and the oxyanion hole are indicated.

Despite differences in size and low primary sequence identity, the lipases show very similar structural folding patterns. This structure is an α/β hydrolase fold, and it is common also to many other hydrolases, such as esterases, proteases, dehalogenases, epoxide hydrolases, and peroxidases [32]. As shown in Figure 3, the canonical α/β hydrolase fold is characterized by the presence of a central β-pleated sheet that contains eight parallel β-strands, with the exception of β2 strand, which is antiparallel with respect to the others. The β3 to β8 strands are connected by α helices, which pack on the two sides of the central β sheet [33].

The active site of the α/β hydrolases consists of a highly conserved catalytic triad: one nucleophilic residue (*i.e.*, serine, cysteine, aspartic acid), one catalytic acidic residue (*i.e.*, aspartic acid, glutamic acid), and one histidine, which acts as a general base catalytic residue. These are always in this order in the amino-acid sequence, which is different from that observed for other proteins that carry the catalytic triad. In the lipases, the nucleophilic residue is always a serine, which is located in the so-called “nucleophilic elbow”, which is identified by the highly conserved pentapeptide GXSXG (Figure 3). The catalytic acidic residue is located after the β6 or β7 strand of the central β sheet, and it is hydrogen-bonded to the catalytic histidine that is located in a loop after the β8 strand of the α/β hydrolase fold (Figure 3). The active site of the lipase also contains a large, hydrophobic scissile FA binding site that accommodates the acyl chain of the ester linkage that is to be hydrolyzed. Additional binding pockets for the other acyl chains of the substrate also frequently contribute to maintaining the substrate anchored to the pentapeptide site residues [34].

Another important feature of the lipases is the oxyanion hole (Figure 3), which is the “pocket” that promotes the stabilization of the negatively charged intermediate generated during the ester-bond hydrolysis. The oxyanion hole consists of two residues, one of which always follows the nucleophilic residue, whereas the other one has a variable position. In the GX class of lipases, the positionally variable residue of the oxyanion hole is the C-terminal neighbour X of a conserved glycine residue that contacts the nucleophilic elbow. In the GGGX class, this oxyanion hole residue is shifted by one position toward the C-terminus, as compared to the GX type, and is a glycine residue that is followed by a conserved hydrophobic residue X. In the Y class, the positionally variable oxyanion hole residue is a tyrosine [35].

Most lipases are characterized by inclusion in their structure of a mobile domain, known as the “lid”, which consists of a single helix, two helices, or a loop region. In the absence of a lipid–water interface, the lid covers the active site, while the presence of an interface results in a rearrangement of the conformation of the enzyme that displaces the lid, thus making the catalytic site accessible to the substrate and the solvent [36]. Lipases from *Bacillus subtilis*, *Bacillus pumilis* and *Bacillus licheniformis*, and a lipase from guinea-pig pancreas, lack the lid domain, and therefore have low molecular weights and do not show interfacial activation [26,37]. In contrast, the lipase from *Pseudomonas* sp. MIS38, which belongs to Family I.3, has two lids: lid1 that represents the widely known lid of the bacterial lipases, and lid2 that is unique to this *Pseudomonas* sp. MIS38 lipase and other Family I.3 lipases [38].

**Figure 4:**
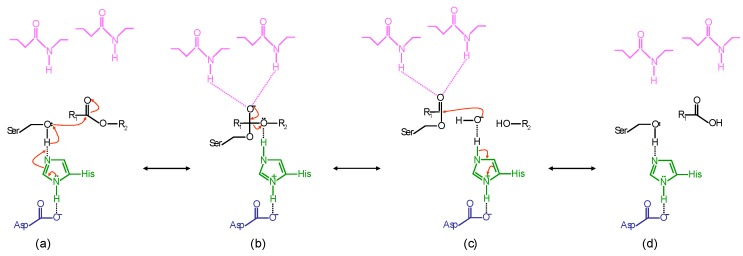
Mechanism of the hydrolysis reaction of ester bonds catalyzed by lipases. Aspartate and histidine are shown in blu and green, respectively; serine, substrate and water are shown in black; the oxyanion hole residues are shown in magenta. (**a**) Nucleophilic attack of the serine hydroxyl on the carbonyl carbon of the susceptible ester bond; (**b**) tetrahedral intermediate; (**c**) acyl-enzyme intermediate, released alcohol and nucleophilic attack by water; (**d**) free enzyme and released acyl product.

The catalysis of lipases follows the classical serine hydrolase mechanism. Substrate hydrolysis starts with the lipid binding and the catalytic serine attack on the carbonyl carbon atom of the susceptible ester bond (Figure 4a). This generates a tetrahedral intermediate that is characterized by the formation of a negative charge on the carbonyl oxygen atom, and the bonding of four atoms to the carbonyl carbon atom arranged as a tetrahedron (Figure 4b). The intermediate is stabilized by two hydrogen bonds between the negatively charged carbonyl oxygen atom and the main-chain -NH groups of the two amino-acid residues of the oxyanion hole. In the lipases of the Y class, one of the two hydrogen bonds is formed with the hydroxyl group of a tyrosine side chain. The nucleophilicity of the serine residue is enhanced by the catalytic histidine, to which a proton from the serine hydroxyl group is transferred. This transfer is facilitated by the catalytic acidic residue, which orients the imidazole ring of the histidine in such a way as to neutralize the charge that is generated on it. Subsequently, the proton is donated to the oxygen atom of the susceptible ester bond, which is thus cleaved, with the consequent release of the alcohol product, while the acyl chain is esterified to the nucleophilic serine (Figure 4c). The next step is the deacylation, in which a water molecule hydrolyzes the covalent bond, with the consequent release of the acyl product and the regeneration of the enzyme (Figure 4d) [34].

### 2.2. Phospholipases

#### 2.2.1. Reactions Catalyzed by Phospholipases

**Figure 5:**
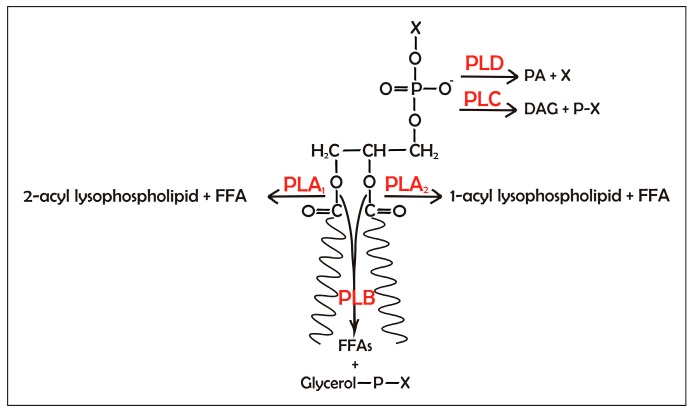
Reactions catalyzed by the phospholipases. The black arrows for phospholipases A_1_, A_2_, B, C, and D (PLA_1_, PLA_2_, PLB, PLC, and PLD) indicate their site of hydrolysis. PLA_1_ and PLA_2_ hydrolyze the *sn*-1 and *sn*-2 acyl ester bonds, respectively, of the phospholipid. PLB sequentially removes the two fatty acids from the phospholipid. PLC hydrolyzes the glycerophosphate bond, and PLD cleaves the terminal phosphodiesteric bond of the phospholipid. DAG, diacylglycerol; FFA, free fatty acid; PA, phosphatidic acid; P-X, phosphorylated head-group; X, head-group. X can be choline, ethanolamine, glycerol, inositol or serine.

Phospholipases are hydrolytic enzymes that act on phospholipids and split them into different products. Similar to lipases, phospholipases are activated by the presence of an interface between an aqueous and an organic phase. Phospholipases are classified based on their substrate cleavage site (Figure 5).

The phospholipases A_1_ (PLA_1_s) and A_2_ (PLA_2_s) catalyze the hydrolysis of the ester bond at the *sn*-1 and *sn*-2 positions of the phospholipids, respectively, thus producing a FFA and 2-acyl lysophospholipid or 1-acyl lysophospholipid, respectively. For the phospholipases that show low selectivity for the *sn*-1 and *sn*-2 positions, the term PLA is used. The phospholipases B (PLBs) can hydrolyze both of the FAs esterified at the *sn*-1 or *sn*-2 position of the phospholipid. The phospholipases C (PLCs) and phospholipases D (PLDs) are phosphodiesterases. PLC cleaves the glycerophosphate bond, thus releasing DAG and the phosphorylated head-group, while PLD cleaves the terminal phosphodiesteric bond, thus releasing phosphatidic acid (PA) along with the head-group [39].

As for the lipases, the use of organic media with low *a*_w_ makes it possible to reverse some of these reactions and to synthesize phospholipids using phospholipases as catalysts. So, PLA_1_, PLA_2_, and PLB can incorporate FFAs into lysophospholipids by acyltransferase and transacylase activities, and PLD can catalyze the transphosphatidylation reaction in which the phosphatidyl group of the phospholipid is transferred to an alcohol, to give the phosphatidyl alcohol [39].

#### 2.2.2. Classification of the Phospholipases

As shown in Table 3, PLA_1_s are ubiquitous enzymes in animals, plants, yeast and fungi, protozoa and bacteria. In mammals, nine PLA_1_s have been identified: six are extracellular and three are intracellular PLA_1_s enzymes. These two groups share no sequence homology and apparently have distinct functions [40]. The six extracellular PLA_1_s belong to the pancreatic lipase gene family, and include hepatic lipase, endothelial lipase, and PLRP2, which also hydrolyze TAGs (see Section 2.1.2 and Table 1), and three specific PLA_1_s, the phosphatidylserine-specific PLA_1_ (PS-PLA_1_), the membrane-associated PA-specific PLA_1_α (mPA-PLA_1_α), and mPA-PLA_1_β [16]. The three intracellular PLA_1_s are PA-PLA_1_, p125, and KIAA0725p, and they are all PA-specific PLA_1_s that are implicated in vesicular transport [40]. In plants, only a few PLA_1_s have been identified, the most important of which is the PLA_1_ identified in *Arabidopsis thaliana* that is called DAD1 (Defective in Anther Dehiscence 1), which is an enzyme that is involved in jasmonic acid biosynthesis [41]. PLA_1_s have also been identified in yeasts and fungi, bacteria and a protozoan [39,40,42].

Currently, the PLA_2_ superfamily consists of several groups of enzymes that are classified into five different classes: the secretory Ca^2+^-dependent PLA_2_s (sPLA_2_s), the cytosolic, Ca^2+^-dependent PLA_2_s (cPLA_2_s), the cytosolic, Ca^2+^-independent PLA_2_s (iPLA_2_s), the platelet-activating-factor acylhydrolases (PAF-AHs), and the lysosomal PLA_2_s (L-PLA_2_s) (Table 3) [43]. The sPLA_2_s (14–18 kDa) have been identified in sources as diverse as animal venoms, mammalian tissues, plants, yeasts and fungi, bacteria and a parvovirus [43,44,45,46]. The cPLA_2_s (61–114 kDa) and iPLA_2_s (28–146 kDa) have been identified in human and mouse. PAF-AHs and L-PLA_2_s have been identified in different mammalian sources [43]. Patatin-like phospholipases, that show PLA_2_ activity, have also been identified in plants and yeasts [45,47,48].

**Table 3:** Classification of phospholipases.

Type	Genera	Organism	Name	Localization	Refs.
PLA_1_	Animal	*Rattus norvegicus*	PS-PLA_1_	Secreted (platelet and various tissues)	[16,40]
		*Homo sapiens*	mPA-PLA_1_α	Secreted (various tissues)	[16,40]
		*Homo sapiens*	mPA-PLA_1_β	Membrane-bound (reproductive tissues)	[16,40]
		*Bos taurus*	PA-PLA_1_	Cytosolic (testis, brain)	[16,40]
		*Mus musculus*	p125	Cytosolic (ubiquitus)	[16,40]
		*Homo sapiens*	KIAA0725p	Cytosolic (ubiquitus)	[16,40]
	Plant	*Arabidopsis thaliana*	DAD1	Chloroplastic (anther)	[41]
	Yeast and fungi	*Aspergillus oryzae*	AoPLA_1_	Secreted	[40]
		*Thermomyces lanuginosus*	PLA_1_	n.d.	[40]
	Protozoa	*Trypanosoma brucei*	TbPLA_1_	Cytosolic	[40]
	Bacteria	*Serratia* spp.	PhlA, PlaA	Secreted	[39,40]
		*Yersinia enterocolitica*	YplA	Secreted	[39,40]
		*Streptomyces alboflavus*	SaPLA_1_	Secreted	[42]
		*Escherichia coli*	OMPLA	Outer membrane-bound	[39,40]
PLA_2_	Animal	Human, porcine, viper and snake, rat, murine, bee, lizard, scorpion, mollusc	sPLA_2_ (Groups IA,IB, IIA, IIB, IIC, IID, IIE, IIF, III, V, X, XII)	Secreted, cytosolic (venoms, synovial fluid, pancreatic juice, various mammalian tissues)	[43]
		Human and murine	cPLA_2_ (Groups IVA-IVF)	Cytosolic (various tissues)	[43]
		Human and murine	iPLA_2_ (Groups VIA-VIF)	Cytosolic (various tissues)	[43]
		Human, murine, porcine, bovine	PAF-AH (Groups VII and VIII)	Secreted, cytosolic (various tissues)	[43]
		Human, murine, bovine	L-PLA_2_ (Group XV)	Secreted, cytosolic (various tissues)	[43]
	Plant	*Arabidopsis thaliana*, carnation, castor bean, citrus, durum wheat, elm, rice, soybean, tobacco	sPLA_2_ (Groups XIA and XIB)	Secreted, cytosolic (various tissues)	[44]
		*Arabidopsis thaliana*, rape, broad bean, cucumber, pepper, potato, tobacco, tomato	Patatin-like phospholipase	Cytosolic (various tissues)	[47,48]
	Yeast and fungi	*Tuber borchii*, *Helicosporum* sp., *Aspergillus oryzae*, *Gibberella zeae*, *Magnaporthe grisea*, *Neurospora crassa*	sPLA_2_ (Group XIV)	n.d.	[45]
		*Aspergillus fumigatus*, *Aspergillus nidulans*, *Aspergillus oryzae*, *Neurospora crassa*	cPLA_2_ (Group IVC)	n.d.	[45]
		*Candida albicans*, *Saccaromyces cerevisiae*	Patatin-like phospholipase	n.d.	[45]
	Bacteria	*Streptomyces coelicolor*, *Streptomyces violaceoruber*	sPLA_2_ (Group XIV)	n.d.	[45]
	Parvovirus	Different species	sPLA_2_ (Group XIII)	n.d.	[46]
PLB	Animal	Human, guinea pig, rat, rabbit, bee, snake	PLB	Secreted, cytosolic (epidermis, enterocytes and neutrophils, venoms)	[49]
	Plant	Broad bean, ricin bean	PLB-like	Cytosolic (leaves)	[50,51]
	Yeast and fungi	*Candida albicans*	CaPlb1-5	Secreted, cytosolic	[52]
		*Cryptococcus neoformans*	CnPlb1	Secreted, cytosolic	[52]
		*Cryptococcus gattii*	CgPlb1	Secreted, cytosolic	[52]
		*Saccaromyces cerevisiae*	PLB1-PLB3	Secreted, cytosolic	[52]
	Amoeba	*Dictyostelium discoideum*	PLB	Cytosolic	[53]
	Bacteria	*Pseudomonas fluorescens*	Pf-PLB	Secreted	[54]
		*Bacillus subtilis*	LipC	Spore coat-bound	[55]
		*Streptomyces* sp.	PLB_684_	Secreted	[56]
PLC	Animal	Mammals	PI-PLC-β, PI-PLC-γ, PI-PLC-δ, PI- PLC-ξ, PI-PLC-η, PI-PLC-like	Cytosolic, nuclear, plasma membrane-bound (various tissues)	[57]
		Mammals	PC-PLC	Cytosolic (erythrocytes, lymphocytes, muscular tissue, adipose tissue, nervous system)	[58]
	Plant	*Arabidopsis thaliana*, bean, cedar, cereals, *Lilium*, moss, potato, soybean, tobacco, tomato	PI-PLC	Cytosolic (various tissues)	[59]
		*Arabidopsis thaliana*,avocado, peanut, rape, rice, soybean, tomato, wheat	NPC	Cytosolic (various tissues)	[60]
	Yeast and fungi	*Cryptococcus neoformans*	PI-PLC (CnPLC1-CnPLC2)	Secreted, cytosolic	[52]
		*Candida albicans*	PI-PLC (CaPLC1-CaPLC3)	Secreted, cytosolic	[52]
	Bacteria	*Listeria monocytogenes*, *Clostridium perfringens*	PI-PLC	Secreted, cytosolic	[52]
		*Bacillus cereus*, *Listeria monocytogenes*, *Clostridium* spp., *Pseudomonas aeruginosa*, *Pseudomonas cepacia*, *Burkholderia pseudomallei*, *Legionella pneumophila*	PC-PLC	Secreted, cytosolic, membrane-bound	[60]
PLD	Animal	Mammals	PLD1, PLD2, PLD3	Secreted, cytosolic, membrane-bound (various tissues)	[61]
		Mammals	mitoPLD	Mitochondrial (various tissues)	[61]
	Plant	*Arabidopsis thaliana,* cabbage, castor bean, grape, *Jatropha curcas*, mustard, peanut, poplar, poppy, strawberry, rice, sunflower, tobacco, tomato	C2-PLD (PLDα, PLDβ, PLDγ, PLDδ, PLDε), PXPH-PLD (PLDζ1, PLDζ2)	Secreted, cytosolic, membrane-bound (various tissues)	[61,62]
	Yeast and fungi	*Saccaromyces cerevisiae*	PLD1 (Spo14), PLD2	Secreted, cytosolic	[61,63]
		*Candida albicans*, *Schizosaccaromyces pombe*	Spo-like		[61]
	Bacteria	*Acinetobacter baumanii*	Act bau PLD	Secreted, cytosolic, periplasmic	[61]
		*Escherichia coli*	Bfil	Secreted, cytosolic, periplasmic	[61]
		*Neisseria gonorrhoeae*	NgPLD	Secreted, cytosolic, periplasmic	[61]
		*Yersinia pestis*	YMT	Secreted, cytosolic, periplasmic	[61]
		*Chlamydia trachomatis*	pz PLDs	Secreted, cytosolic, periplasmic	[61]
		*Pseudomonas aeruginosa*	PLDa	Secreted, cytosolic, periplasmic	[61]
		*Streptomyces* PMF	PMF PLD	Secreted, cytosolic, periplasmic	[61]

The PLBs have been widely demonstrated in both animal and microbial sources (Table 3). For the animal sources, PLBs have been detected in mammalian tissues and animal venoms [49]. PLBs were also identified in a social amoeba [53], bacteria [54,55,56], nonpathogenic and pathogenic yeasts and fungi [52]; in these latter, PLBs appear to act as virulence factors. A PLB-like activity has also been detected in plants [50,51].

The PLCs in living systems can generally be divided into phosphatidylinositol-specific PLCs (PI-PLCs) and phosphatidylcholine-specific PLCs (PC-PLCs) (Table 3). In mammals, there are a total of 13 PI-PLC isozymes that are subdivided into six classes (PI-PLC-β, PI-PLC-γ, PI-PLC-δ, PI-PLC-ξ, PI-PLC-η, and PI-PLC-like); their roles in signal transduction have been widely investigated [57]. In plants, PI-PLCs closely related to mammalian PI-PLC-δ have been identified, and evidence has been reported for their involvement in the regulation of growth, development and stress responses [64]. PI-PLCs have also been identified in different pathogenic bacteria, yeasts and fungi, in which these enzymes act as pathogenicity factors that typically confer virulence [52]. For the PC-PLCs, the major gap in our knowledge of these important enzymes has arisen because the mammalian PC-PLCs have not been cloned and their sequences are not known. However, PC-PLC activity has been detected in different animal sources, where these enzymes are implicated in several cellular signaling pathways [58]. In plants, non-specific PLCs (NPCs) have been discovered, homologous to bacterial PC-PLCs. In particular, in *A. thaliana* evidence has been provided for their involvement in hormonal and stress signaling [60]. In bacteria, the PC-PLCs have been well characterized in pathogenic bacteria, in which these enzymes act as potent toxins with hemolytic properties, and in nonpathogenic bacteria in which they appear to be involved in lipid modeling under phosphate-limiting conditions [60].

The PLDs belong to a superfamily of signaling enzymes that most commonly generate the lipid second messenger PA. This class of phospholipases was first identified in plants; subsequently, a number of PLD isoforms were detected and characterized in mammals, bacteria and yeasts (Table 3) [61]. Plant PLDs can be divided into two subclasses: the C2-PLDs and the PXPH-PLDs. The C2-PLDs have an N-terminal C2 Ca^2+^-binding domain; these enzymes are involved in lipid metabolism, signal transduction, and membrane trafficking. The PXPH-PLDs have N-terminal phox homology (PX) and pleckstrin homology (PH) domains, which are important for specific lipid interactions; a role in environmental stress responses has been postulated for this subclass of PLDs. In mammals, three isoforms of PLD have been identified (PLD1-PLD3). A fourth PLD isoform, called mitoPLD, has also been described, which has an N-terminal mitochondrial localization sequence. The PLD enzymes are not commonly expressed among bacteria, compared to other phospholipases, but members of the PLD superfamily have been identified in many nonpathogenic and pathogenic bacteria. In these latter, the PLD enzymes act as toxins, and can thus lead to severe cell damage. In yeast, PLD1 (Spo14) has been identified in *Saccaromyces cerevisiae* and, subsequently, Spo14-like enzymes were identified in other yeasts. Yeast PLD1s are involved in yeast sporulation, vesicular trafficking, mating, and virulence for the pathogenic species [61]. In addition, a biochemically distinct enzyme, PLD2, has been described in *S. cerevisiae* that is probably involved in the initiation of mitotic cell division in yeast [63].

#### 2.2.3. Structural Features and Catalytic Mechanisms of the Phospholipases

As members of the pancreatic lipase family, mammalian PLA_1_s show structural similarities with lipases and have the typical Ser–His–Asp triad, with the serine residue lying in a GXSXG motif. However, compared to lipases, PLA_1_s have shorter surface loops (β5 loop, β9 loop and lid domain), which appear to be responsible for their substrate specificity [16]. The catalytic triad and the GXSXG motifs have also been identified in DAD1 of *A. thaliana* [41]. For bacteria, outer-membrane PLA (OMPLA) has an Asn instead of an Asp residue in the catalytic triad [39], whereas the cytosolic PLA_1_s have a Ser–His dyad [42]. For all of the PLA_1_s, the catalysis follows the classical serine hydrolase mechanism, as already described above for the lipases (see Section 2.1.3).

The PLA_2_ classes differ strongly from each other with respect to their structural features. sPLA_2_s contain a catalytic site, a Ca^2+^-binding loop, and a signal peptide for secretion, as well as twelve cysteine residues that form six intramolecular disulfide bridges, with one or two variable disulfide bridges [43]. The catalytic mechanism of the sPLA_2_s uses the histidine of the His-Asp dyad as the primary catalytic residue. Aided by the aspartate residue, this histidine residue polarizes a molecule of water, which then attacks the ester bond at the *sn*-2 position of the phospholipid. Ca^2+^ has a direct role in this catalysis, as it stabilizes the transition-state intermediate. cPLA_2_s are composed of a Ca^2+^-dependent lipid binding C2 domain and a catalytic α/β hydrolase domain, whereas the iPLA_2_s are composed of 7–8 ankyrin repeats, a linker region, and a catalytic domain. The activities of the cPLA_2_s and iPLA_2_s involve a catalytic serine that is located in the middle of the GXSXG consensus sequence, and which in some cases is part of a Ser-Asp dyad. In the cPLA_2_s, Ca^2+^ is required to promote the enzyme-membrane interaction. The PAF-AHs and the L-PLA_2_s are serine hydrolases that, as with the cPLA_2_s and iPLA_2_s, have the GXSXG motif, although they show the catalytic Ser–Asp–His triad instead of the dyad [43].

The PLBs are generally glycosylated ecto-phospholipases that have a signal peptide for secretion at their N-terminus, an extracellular domain, and a C-terminal hydrophobic sequence that anchors the protein in the membrane. In animal PLBs, the large extracellular domain consists of four tandem homologous repeats (I–IV), where repeats II to IV contain several GDSL and GXSXG motifs, although only the serine residue in domain II is responsible for the enzyme activity [65]. Bacterial PLBs belong to the SGNH hydrolase family that have four strictly conserved residues, Ser–Gly–Asn–His, in four conserved blocks, I–III and V. The catalytic serine residue relies at the N-terminus and it is part of a putative Ser–His diad (e.g., in PLB_684_ from *Streptomices* sp.) [56] or a Ser–Asp–His triad (e.g., in LipC from *B. subtilis*) [55]. The GXSXG consensus sequence and the catalytic triad that comprises Arg, Ser and Asp have been identified in fungal PLBs [66,67]. The PLB from the social amoeba *Dictyostelium discoideum* lacks the GXSXG motif, but has an NSGTYN(S/N)Q sequence that contains a serine residue that might be involved in catalysis [53].

Mammalian PI-PLCs consist of four domains: from the N-terminus to the C-terminus, they have a PH domain that is involved in the binding of the PI phosphates, an EF-hand domain that appears to be responsible for Ca^2+^-sensing, a catalytic domain that accommodates both the Ca^2+^ and the substrate head-group, and a C2 domain that is responsible for the Ca^2+^-dependent targeting of PLCs to membranes [68]. The PH domain is absent in plant PI-PLCs [69]. Bacterial PI-PLC folds as a single domain structure that is similar to the catalytic domain of the mammalian PLCs [70]. A two-step catalytic mechanism that involves Ca^2+^ as a cofactor has been proposed for both mammalian and bacterial PI-PLCs. The reaction proceeds by general acid/base catalysis. The first phosphotransfer step generates a stable cyclic phosphodiester intermediate and, in the second step, phosphohydrolysis of the cyclic intermediate generates the acyclic inositol phosphate [68].

For the PC-PLCs, to date there remains no information on the structural features of the mammalian PC-PLCs. On the contrary, the molecular structures of the bacterial PC-PLCs have been well characterized. These have a signal peptide for secretion, the active site at the N-terminus, and a C2-like PLAT (polycystin-1, lipoxygenase, α-toxin) domain at the C-terminus, which appears to be responsible for the Ca^2+^-dependent binding of the enzyme to the membrane. Toxic PC-PLCs require Zn^2+^ for their catalysis, whereas the nontoxic PC-PLCs do not [60]. NPC from plants have a phosphoesterase domain that is flanked by two highly conserved motifs, with two other conserved motifs in the middle of the domain. The greater part of these enzymes contains a putative signal peptide at the N-terminus. For their catalytic mechanism, evidence has been reported that both eukaryotic and bacterial PC-PLCs share a common ping-pong reaction mechanism, with an intermediate in which the substrate phosphate group is covalently bound to the nucleophilic amino acid from the active site [60].

All of the PLDs have an active site that consists of four conserved amino-acid motifs, from I to IV. Motifs II and IV contain the conserved sequence HXKXXXXDX6GSXN, which is generally abbreviated to HKD, and which is responsible for the PLD catalytic activity [71]. Both mammalian and plant PLDs have other highly conserved regions. These include the N-terminal PX and PH domains, both of which mediate PLD-phospholipid interactions. In addition, the PH domain regulates the PLD localization to the membranes [71]. There is also a C2 domain in the N-terminal region of plant PLDs, which is responsible for mediating Ca^2+^-dependent phospholipid binding to the enzyme [69]. The enzymes of the PLD superfamily are proposed to follow a similar reaction mechanism. Initially, the phospholipid enters the active site of the enzyme, where in its protonated form, the histidine of the HKD motifs is responsible for the nucleophilic attack on the substrate phosphorus. This leads to the generation of the free head-group of the phospholipid, and the formation of a phosphatidylated enzyme intermediate. Finally, the histidine re-acquires a proton from a water molecule, which leaves the hydroxyl group to the intermediate and releases PA [61].

## 3. Lipase and Phospholipase in Biocatalysis

The hydrolytic activity of the lipase has been widely used for industrial applications. This reaction is generally carried out in environments that contain a minimum of two distinct phases, where all of the reactants are partitioned between these phases. The hydrolytic activity of lipases is used extensively in the formulation of laundry detergents for the removal of fat-containing and oil-containing stains, in the treatment of wastewaters that contain fats and oils, in the leather industry for removal of fats from hides and skins, and in the paper industry for removal of fats from wood. TAG hydrolysis is used in the food industry for the release of FFAs, which develop flavored products, as well as for the production of DAGs and MAGs, which are used as emulsifiers [33,72].

The synthetic potential of lipases has also been widely exploited in lipid biocatalysis. Both the esterification and the transesterification reactions can occur in organic media, and can be used to produce commercially relevant esters. However, transesterification has some advantages compared to direct synthesis, as no water molecules are formed in the reaction and the enzyme stability is usually higher under these conditions [73]. One particularly commercially relevant category of esters produced by lipases is that of the FA alkyl esters (FAAEs), which are also called biodiesels, and which have become an important renewable fuel source. Other examples of esters produced by lipases for commercial purposes are the MAGs, DAGs and sugar esters that are used as emulsifiers, and the structural lipids that are used as nutraceuticals [33].

Lipases are also increasingly used for kinetic resolution of chiral compounds that serve as building blocks for the synthesis of chiral pharmaceuticals, pesticides and agrochemicals. Resolution occurs through the transesterification reaction; thus, in the presence of a suitable acyl donor, one enantiomer of the racemic mixture is selectively transferred to the corresponding ester, which leaves the second unreacted enantiomer in a pure form [74].

Phospholipases are used industrially to modify phospholipids. These modifications mainly include the hydrolysis of phospholipids, the synthesis of specific phospholipids, and the conversion from one phospholipid class to another. The hydrolysis of FFAs from the glycerol backbone of phospholipids is carried out by using PLA_1_s, PLA_2_s and PLBs, as well as lipases; in this last case, the reaction is carried out using specific 1,3-lipases, which are also specific for the *sn*-1 position of phospholipids. This reaction is of relevance in oil degumming and in dairy and bakery industry applications [9,75]. Also, hydrolysis of the phosphorylated head-group by PLC is of interest in oil degumming [75].

In the synthesis of new phospholipids, one of the two FAs that are esterified to the glycerol backbone is changed by a two-step process of hydrolysis and esterification, or a one-step process of transesterification. While the exchange at the *sn*-2 position can be carried out using PLA_2_s, for the exchange at the *sn*-1 position, 1,3-lipases are often preferred to PLA_1_s. This exchange is of particular relevance for the industrial production of nutraceutical phospholipids. 

For the conversion of one phospholipid class to another, PLD-catalyzed transphosphatidylation is used. This is of particular interest for the industrial production of unnatural or low-abundance phospholipids with nutraceutical or pharmacological properties [75].

### 3.1. Source of Lipases and Phospholipases for Biocatalysis

As indicated above, lipases and phospholipases are ubiquitous in nature and are produced by animals, plants and microorganisms. Nevertheless, due to their ease of manipulation, their unlimited supply, and their particular catalytic properties, microbial lipases occupy the prominent position as industrially important biocatalysts. Indeed, according to the list of enzymes compiled by the Association of Manufactures and Formulators of Enzymes Products (AMFEP, http://www.amfep.org/content/list-enzymes) [76] and updated in May 2015, the main sources of lipases and phospholipases used in industrial processes are fungi, followed by yeasts, bacteria and animals. To date, among the known lipases and phospholipases from plant sources, only cabbage PLD has been successfully applied to industrial processes. Nevertheless, in recent years, the search for new sources of lipases and phospholipases has been intensified, and some promising plant enzymes have been investigated for their potential use as biocatalysts.

#### 3.1.1. Animal Sources

The animal lipases used in industrial processes include pancreatic and pregastric lipases. Lipase extracted from pancreatic juice provided the first lipase used for industrial applications. In 1856, Claude Bernard discovered a lipase activity in the pancreatic juice that hydrolyzed insoluble oil droplets and converted them into soluble products. Pancreatic lipases and pregastric lipases purified from calf, goat and lamb gullets are used in the acceleration of cheese ripening and flavor generation, as well as in the lipolysis of butter, fat and cream [77].

With regard to the animal phospholipases, the only ones used in industrial applications are PLA_2_s [9]. Commercial PLA_2_ is produced from porcine pancreas, and it is used industrially for the production of lysolecithin, which is used as an emulsifier and antifungal agent in foods. Porcine PLA_2_ is also suitable for enzymatic degumming of edible oils [75]. Recently, the starfish *Asterina pectinifera* [78] and the common stingray *Dasyatis pastinaca* [79,80] have been reported as potential new sources of PLA_2_ with potential application as biocatalysts, thus providing alternatives to porcine pancreatic PLA_2_.

#### 3.1.2. Plant Sources

Lipases from oilseeds have the particular characteristic that they are very active when the *a*_w_ in the environment is very low, thus making these enzymes suitable to work as biocatalysts in various organic solvents [21,22]. Within cereal seeds, the lipases from rice, wheat, barley, oat, and maize have been tested for their use as biocatalysts, and most of these are resistant to high temperatures and alkali conditions [81,82]. Interestingly, Chong and coworkers [83] successfully developed a method for the biosynthesis of acylglycerols using a delipidated rice bran lipase to esterify palm oil FAs with glycerol. In recent years, the industrial applications of lipases obtained from latex from different rubber trees have been exploited [25]. In particular, *Carica papaya* lipase has emerged as a versatile biocatalyst, and many biotechnological applications have been reported using this enzyme, either as a crude extract or in a partially purified form, such as fats and oils modification, esterification and interesterification reactions in organic media, asymmetric resolution of different chiral acids [84].

For the phospholipases, PLD represents the only type of plant phospholipase with potential applications as a biocatalyst, due to its catalysis of transphosphatidylation reactions [85]. Among plants, the most traditional source of PLD is cabbage (*Savoy cabbage*). Cabbage PLD has been succesfully used for modification of polar head-groups, to produce synthetic or low-abundance phospholipids for nutraceutical and pharmacological purposes [86].

#### 3.1.3. Microbial Sources

Lipases and phospholipases from yeasts and fungi are the most relevant for commercial applications, followed by those produced by bacteria. The most important genera of yeasts and fungi that are exploited for their production of lipases and phospholipases include *Candida*, *Yarrowia*, *Aspergillus*, *Penicillium*, *Rhizopus*, *Rhizomucor*, and *Thermomyces*. In particular, among the yeasts, due to its 1,3-specificity, high enantio-selectivity against secondary alcohols and primary amines, and extraordinary stability in organic solvents and at extreme temperatures, *Candida antarctica* lipase B (CALB) is the most frequently used enzyme in numerous biocatalytic processes, and it has been the subject of a great number of patents [87]). Another industrially relevant lipase from the yeast genus *Candida* is *Candida rugosa* lipase (CRL), which is commercially available as a mixture of different isoforms, and as this preparation is “Generally Recognized As Safe” (GRAS), it is widely used in the food industry. Among fungi, *Rhizopus oryzae* lipase (ROL), *Rhizomucor miehei* lipase (RML), *Thermomyces lanuginosus* lipase (TLL) and *Fusarium heterosporum* lipase (FHL) are the ones that are typically used in biodiesel production [88], while lipases from *Aspergillus niger*, *Aspergillus oryzae*, *Penicillium roqueforti*, *Penicillium camemberti*, *Penicillium cyclopium* and *Rhizomucor javanicus*, together with ROL and RML, are used in food processing and nutraceutical synthesis [89]. TLL is also widely used as a detergent additive [90]. Among the yeast and fungal phospholipases, PLA_1_s and PLA_2_s from *Fusarium oxysporum,* T. lanuginosus, *A. niger* and *Trichoderma reesei* have been commercialized and are used in the degumming of vegetable oils, while PLA_1_s, PLA_2_s and PLBs from *A. oryzae* and *A. niger* have been largely used in the food industry [9,75]. PLDs from *Actinomycete* strains are also commercially available and are used in industrial processes, because of their high transphosphatidylation and hydrolytic activities [75].

For bacteria, the most important genera that have been investigated for the production of lipases and phospholipases are *Pseudomonas*, *Bacillus* and *Streptomyces*, followed by *Burkholderia*, *Chromobacterium*, *Achromobacter*, *Alcaligenes* and *Arthrobacter*. Of these, a number of bacterial lipases and phospholipases are used in industrial processes, such as biodiesel production, oil degumming, synthesis of nutraceuticals, and in detergent formulations, whereas they have been poorly applied to the food industry. In particular, lipases from *Pseudomonas fluorescens* and *Burkholderia cepacia* and *Bacillus thermocatenulatus* are used in biodiesel production [88], while lipases from *Pseudomonas mendocina*, *Pseudomonas alcaligenes*, *Pseudomonas glumae* and *B. cepacia* are used as detergent additives [91]. Among the bacterial phospholipases, PLA_2_, PLB and PLC produced from *Streptomyces violaceoruber*, *P. fluorescens* and *Bacillus anthracis*-like strain are used in degumming of vegetalbe oils [9]. PLDs from *Streptomyces* strains are used in the food industry and in the synthesis of nutraceuticals [75].

### 3.2. Properties of Lipases and Phospholipases Suitable for Biocatalysis

Each industrial application requires unique enzymes with respect to their specific properties, and as indicated above, this is one of the main reasons for the enormous biotechnological potential of the microbial lipases and phospholipases. The widening application of microbial lipases in biotechnology has necessitated the exploration of their biodiversity in the continuing research for novel enzymatic variants that might be more beneficial for industrial uses. This has included, for instance, the screening of bacterial strains that can be recovered directly from environmental samples, which has allowed the identification of a great number of new enzymatic variants. Thus, new lipases and phospholipases have been identified that show higher substrate specificity or enantio-selectivity, higher stability with extreme temperatures, pHs or organic solvents, and tolerance to proteolysis.

#### 3.2.1. Substrate Specificity

Substrate specificity of lipases is crucial to their application for industrial purposes. As far as the type of FAs, different specificities have been observed among microbial lipases. For instance, ROL and lipase from *Penicillium caseicolum* show preference for short-chain FAs, CRL and lipases from *A. niger* and *Rhizopus* are specific for medium-chain FAs, whereas lipases from *Candida curvata* and *Candida parapsilosis* show preference for long-chain polyunsaturated FAs (PUFAs). As far as class specificity, examples are the lipase from *P. cyclopium*, that shows preference for MAGs, the lipases from *P. camemberti*, *Fusarium* sp. YM-30 and *A. oryzae* that specifically hydrolize DAGs and MAGs but not TAGs, and the RML that hydrolyzes TAGs at higher rate than DAGs and MAGs [72,92]. As far as the regio-specificity, lipases isolated from the yeast *C. rugosa*, the fungi *Geothricum candidum* and *Penicillium expansum*, and the bacteria *Pseudomonas*, *Burholderia* and *Alcaligenes* are nonspecific and completely hydrolyze TAGs into FFAs and glycerol; on the contrary, pancreatic lipase, as well as lipases from the fungi *A. niger*, *Rhizopus arrhizus*, *R. miehei* and *R. oryzae* release FFAs regio-specifically from the *sn*-1 and *sn*-3 positions [72,92]. A lipase has also been identified in *Geotrichum* sp. FO401B that hydrolizes TAGs selectively at the central *sn*-2 position [93]. Interestingly, CALB changes its regio-specificity, as it acts on 1,3- and 2-positions of TAGs at the same rate in alcoholysis with low amount of methanol and ethanol, but shows preference for the 1,3-position in the presence of excess amounts of these alcohols [72]. Many lipases from *Pseudomonas*, *Burkholderia*, *Serratia marcescens*, *C. rugosa* and *C. antarctica* are able to discriminate between enantiomers of chiral compounds and have been, therefore, successfully used to synthesize optically active compounds, that are important intermediates in the synthesis of drugs, herbicides and flavors [72].

As far as the phospholipases, the regio-specificity of this class of enzymes for the *sn*-1, *sn*-2 or *sn*-3 position of the glycerol backbone depends exclusively on the type of phospholipase employed, as each phospholipase has a specific site of action. As far as the substrate specificity of phospholipases, most of the microbial enzymes, once secreted outside the cell, are responsible for cytolytic activity of the producing microorganism. This activity requires broad substrate specificity; so, generally, phospholipases from microbial sources share as major substrate PC, which is the most abundant among the phospholipids, but they can also act on the other phospholipids and lysophospholipids without showing a particular preference [94]. This is a desirable feature for their industrial applications, such as food industry and oil degumming, in which these enzymes are largely employed (see Section 5.2 and Section 5.3). Moreover, the high ability of phospholipases to use PC as substrate is of particular interest for the industrial production of unnatural phospholipids or phospholipids that are not abundant in natural sources, as in both cases PC, for its natural abundance, is the most commonly used starting material [75].

#### 3.2.2. Stability in Organic Solvents

As indicated above, for industrial purposes, the use of lipases and phospholipases in organic solvents is advantageous, as it shifts the equilibrium of the reaction towards ester synthesis. Moreover, the use of organic solvents increases the rigidity of these enzymes, thus improving their substrate specificity and their thermal stability. Additional advantages here are the improvement in the solubility of substrates, the elimination of side reactions, and the prevention of microbial contamination [95].

Recently, different genera of organic-solvent-tolerant bacteria have been isolated and screened for their lipolytic activities. In particular, different strains from *Pseudomonas* sp. can grow on organic media and their organic-solvent-tolerant lipases have been isolated. Thus, organic-solvent stable lipases have been purified from *Pseudomonas aeruginosa* [96,97,98] and *P. fluorescens* [99,100]. Also, the strain S5 of *Pseudomonas* sp. isolated from a soil sample has been shown to produce a lipase that has activity in the presence of cyclohexane and *n*-hexane that is more than 2-fold and 4-fold higher, respectively, than in the absence of these organic solvents [101]. In addition to bacterial strains from *Pseudomonas* sp., strains from *Bacillus* sp. have also been isolated that have organic-solvent-tolerant lipases. Notably, the lipases produced by *Bacillus* sp. strain 42 [102] and *Bacillus sphaericus* 205y [103] have been shown to be stable in different organic media, with *B. sphaericus* 205y even activated in the presence of *n*-hexane and *p*-xylene, by 3.5-fold and 2.9-fold, respectively.

For phospholipases, a PLB that shows remarkable organic-solvent tolerance has been purified from *Thermotoga lettingae* (TLPLB). This TLPLB was shown to be more stable in non-polar than polar organic solvents. Indeed, after incubation in hydrophobic organic solvents, TLPLB retained 91% to 161% of its phospholipase activity, while in hydrophilic solvents its residual activity was only 10% to 38% [104]. Recently, the two PLA_2_s from pancreas [79] and intestine [80] of the common stingray were shown to retain 100% of their activity in incubations with polar organic solvents; increased activity was even observed in the presence of ethanol and acetonitrile (by 12% to 23%).

#### 3.2.3. Tolerance to High and Low Temperatures

High operation temperatures are clearly advantageous in industrial processes, as they are helpful to raise the reaction rate and the process yield, by increasing substrate and product solubility and favoring the equilibrium displacement in endothermic reactions. In addition, elevated temperatures reduce microbial contamination by mesophilic bacteria [105]. Indeed, a number of applications exist that are favored at relatively high temperatures, but it should be taken into consideration that this implies supplementary economic charge and leads to environmental problems. Moreover, high temperatures might lead to thermal denaturation, which represents a common cause of enzyme deactivation. Thermostable enzymes are generally produced by thermophilic bacterial strains from different exotic ecological zones and volcanic areas, where they are adapted to grow at temperatures between 60 and 100 °C, and secrete enzymes that are stable at these temperatures to support their physiological processes.

As a result, thermostable lipases that are active at temperatures between 50 and 75 °C have been purified from several thermophilic strains from *Bacillus* and *Geobacillus* spp. [106,107,108]. Of particular relevance here, there is the very recent identification of a strain of *Geobacillus kaue* and four strains of *Geobacillus kaustophilus* that show optimal activities at 80 °C, which are almost completely maintained even at 90 °C [109]. Thermotolerant lipases have also been isolated from other bacterial genera. Two thermostable lipases, LipA and LipB, were isolated from the anaerobic thermophilic bacterium *Thermosyntropha lipolytica*, and the temperature for the maximal activity of these enzymes was around 96 °C, which is the highest temperature for maximal activity among lipases [110]. Among mesophilic bacteria, an extracellular hyper-thermostable lipase has been isolated from a *Pseudomonas* sp. strain, which has an optimal activity at 90 °C and a half-life of more than 13 h at this temperature [111].

For the phospholipases, highly thermostable PLA_2_s have been isolated from the hyper-thermophilic archaea species *Pyrococcus horikoshii* [112] and *Aeropyrum pernix K1* [113]. Both of these PLA_2_s show their optimal activity at 90 °C and retain most of their activity after incubation at temperatures ranging from 80 to 100 °C. As well as their stability in organic solvents, the two PLA_2_s from the common stingray [79,80] and the TLPLB [104] were shown to be also thermotolerant. Indeed, these PLA_2_s showed optimal activity at 40 to 45 °C and maintained 75% to 80% of their maximal activity after incubation at 70 °C; similarly, TLPLB showed its maximal activity at 70 °C, and maintained 50% of this activity at temperatures from 30 to 100 °C. Thermostable PLDs have also been identified in *Streptomyces olivochromogenes* [114] and in plant sources, such as *Brassica juncea* seeds [115] and *Allium sativum* bulbs [116]. These PLDs showed optimal activity at 60 to 70 °C and were active over a broad range of temperatures (30 to 85 °C).

Biocatalysis performed at low temperature by using cold-active enzymes offers important opportunities for environmental and economic benefits through energy saving. In addition, in the food industry, cold-active enzymes represent the enzymes of choice, to avoid alteration of food ingredients, and in the formulation of detergents their addition allows to work at lower washing temperatures. However, despite the enormous advantages of the application of cold-active lipases and phospholipases in biotechnology, studies in this field have not developed as rapidly as in the field of thermotolerant lipases and phospholipases. Cold-active enzymes are produced by psychrophilic and psychrotrophic species that can survive or even thrive in permanently cold habitats, such as the Antarctic and Polar regions, in glaciers and mountain regions, or in deep-sea sediments. Cold-active lipase-producing microbes can also be isolated from soils or waters that reach low temperatures diurnally or seasonally.

Cold-active lipase-producing bacterial strains isolated from such extreme environments belong to the genera *Acinetobacter*, *Aeromonas*, *Colwellia*, *Janibacter*, *Morexella*, *Pseudomonas*, *Pseudoalteromonas*, *Psychrobacter*, *Rhodococcus* and *Stenotrophomonas* [117,118,119,120,121,122,123,124]. All of these enzymes are characterized by low temperatures for optimal activity, which ranges from 4 to 35 °C, with some of them being unstable or inactivated at temperatures greater than 30 to 50 °C. Cold-active lipases that show optimal activities between 20 and 37 °C have also been isolated from psychrotolerant bacteria, such as *P. fluorescens*, *Serratia epidermidis* and *S. marcescens*, isolated from refrigerated or frozen foods [118]. Psychrophilic yeasts are also a good source of cold-active lipases. Yeast species, such as *Candida lipolytica*, *G. candidum* and *P. roqueforti*, isolated from frozen foods have been reported to produce cold-active lipases, although the most widely studied psychrophilic lipase is CALB from a *C. antarctica* strain isolated from the sediment of an Antarctic lake [125]. This enzyme shows its optimal activity at 40 °C, and its stability strongly decreases at higher temperatures [126].

Cold-active phospholipases have been isolated from different bacterial sources. PLA_1_s active at low temperatures have been isolated from a psychrotrophic, glacier and soil bacterium of *Serratia* sp. [127] and from a soil bacterium of *Bacillus cereus* sp. [128]. Both of these PLA_1_s have their optimum activity at 35 °C and retain most of their maximal activity at 10 and 4 °C, respectively. A cold-adapted PLA_1_ has also been isolated from a metagenome library of the tidal flat sediments on the Korean coast [129]. This enzyme, designated MPlaG, showed its optimum activity at 25 °C and retained 39% of this maximal activity at 5 °C, while its activity drastically decreased at temperatures above 25 °C. A cold-active PLD has also been isolated from the psychrophilic bacteria *Shewanella* sp. The enzyme activity was optimal at 40 °C, but it was also stable below 30 °C and relatively labile above 40 °C, with a rapid loss of activity at 60 °C [130].

#### 3.2.4. Tolerance to Alkaline and Acidic pHs

Alkaline enzymes represent attractive catalysts for many industrial applications, particularly in detergent formulation, as a detergent lipase needs to be active and stable in the alkaline (pH 8–11) environments encountered under harsh washing conditions.

Bacterial lipases are mostly alkaline in nature. Alkaline lipases that show optimal activities in the pH range from 9 to 11 have been isolated from strains belonging to *Acinetobacter radioresistens*, *Bacillus* sp., *Choromobacterium viscosum*, *Micrococcus* sp., and different species of the genus *Pseudomonas* [131], as well as from the strains *B. cepacia* ATCC 25609 [132], *Acinetobacter johnsonii* LP28 [119], *Staphylococcus* sp. ESW [133], *Bacillus* sp. LBN 2 [134], and *Bacillus flexus* XJU-1 [135]. Alkaline lipases have also been isolated from the yeasts *Humicola lanuginosa*, *Trichoderma lanuginosus*, *Trichosporon asahii* [131] and, recently, *Rhodosporidium babjevae* BD19 [136].

Among the phospholipases, stability at extreme temperatures is often associated with stability at extreme pHs. Indeed, the two thermostable PLA_2_s from common stingray [79,80], the thermostable PLD_528_ from *S. olivochromogenes* [114], and the metagenome-derived cold-active MPlaG [129] have pH optima at 8.0–9.5 and have been shown to be stable in the alkaline range.

Compared to these alkaline enzymes, information on the acidic enzymes is rare. A few have, however, reported on acidic lipases from both bacteria, including *B. pumilus* and *B. subtilis* [137], *Pseudomonas gessardii* and *P. fluorescens*-like [138,139], and yeasts and fungi, including *A. niger* [140,141] and *Candida viswanathii* [142]. All of these enzymes show highly acidic pH optima that range between pH 1 and 3. The most important application of these acidic lipases is in the flavor industry, as aroma esters are formed in acidic environments, and also in the leather industry for acid bathing of fur [7].

#### 3.2.5. Tolerance to Proteases

Proteases and lipases have a wide range of applications and there are industrial processes that require a combination of these two, such as the detergent industry and bioremediation. So, for these applications, lipases that are stable against digestion by proteases are strictly required. Nevertheless, to date, few proteolysis-resistant lipases have been identified. Zhang and coworkers [143] cloned a lipase gene, *lipS221*, from *Streptomyces fradiae* var. k11 that was found to be stable toward degradation by commercial alkaline and neutral proteases. Also, a *B. cereus* strain has been identified that can produce an alkaline thermostable extracellular lipase that is resistant to trypsin treatment [144]. An economically advantageous alternative in the formulation of commercial enzyme mixtures is represented by the concomitant microbial production of both lipases and proteases, although this is feasible only if the lipase is resistant to proteolytic degradation by the co-produced protease. Here, evidence has been reported of the concomitant production of a protease and a proteolysis-resistant lipase in strains of *S. marcescens* and *P. aeruginosa* [145], *B. licheniformis* [146] and *B. pumilus* [147].

## 4. Heterologous Expression

Similar to the enzymes purified from their natural sources, recombinant enzymes produced for industrial purpouse are also mainly from microbial origins. This is essentially because most microorganisms have a simple genome that has benefited from the recent success of sequencing programs. This has given the opportunity to identify an increasingly greater number of gene sequences that encode new variants of microbial enzymes. So, most of these genes have been cloned and successfully expressed in heterologous microbial hosts.

The enzyme industry has taken advantage of heterologous expression to increase the reproducibility and the yields of the production of enzymes, which are the major obstacles to their use at an industrial scale. Moreover, heterologous expression allows the production of native enzymes that are synthesized by microorganisms that cannot be easily grown by traditional culture-based methods. In this case, a metagenomic approach can be applied that is based on genomic analysis of microorganisms through the extraction of DNA recovered directly from environmental samples, and its cloning in a heterologous host to generate a metagenomic library that can be fully screened to find the enzyme variant with the desired features. Another huge advantage of heterologous expression is the possibility to produce not only natural but also engineered enzymes. Through the manipulation of the gene encoding a specific enzyme, a new sequence is obtained that encodes a new more stable and selective catalyst that can operate with high efficiency under specific processing conditions.

Both purified recombinant enzymes and whole recombinant cells can be used as biocatalysts. The use of a purified enzyme offers several benefits, as it allows the production of an enzyme that is substantially free of undesirable microbial enzymes or metabolites and ensures high yields due to the high concentration of the catalyst. Compared to the purified enzyme, the whole-cell biocatalyst can be much more inexpensively prepared, and because the enzymes in the cells are protected from the external environment, they are generally more stable in the long-term than the free enzymes. However, whole-cell systems require time-consuming work-up because of the large cell volumes needed, and as they provide low productivity and might give rise to undesirable side reactions. Another drawback in the use of whole-cell systems is that sometimes the cell membrane can act as a barrier between the enzyme and the substrates [4,148]. Recently, one of the most exciting developments for whole-cell biocatalysis was the possibility to directly target the recombinant protein on the cell surface of the host organism by fusing it with cell-surface proteins [149]. This strategy allows us to overcome some drawbacks related to the use of the whole-cell system, as it allows the enzyme to react with high-molecular-weight or toxic substrates that cannot be incorporated into the cell, and it prevents enzyme inactivation and product degradation due to undesired intracellular enzymes and metabolites.

For lipases and phospholipases, the production of these enzymes in their native form for industrial applications has several drawbacks. Indeed, to reach high yields of enzyme production, the microorganism has to be grown in the presence of lipids as the carbon source, and as most of the microorganisms that produce lipases and phospholipases are aerobic, a complex culture medium is required for the production of these enzymes, including biomass, water, organic solvent, and air [150]. Moreover, microorganisms, and yeasts and fungi in particular, often produce different lipase and phospholipase isoforms that are characterized by different catalytic behaviors. The purification of a single isoform from a mixture of isoforms that show high amino-acid identity to each other is an expensive and time-consuming process; as a consequence, commercial preparations often include different isoforms, which might result in irreproducible results and undesirable side effects. Thus, the use of heterologous expression systems to produce recombinant lipases and phospholipases might represent an alternative to the extraction of these enzymes from their natural microbial sources. A strategic coalition between heterologous protein expression and protein engineering is thus leading to continuously growing numbers of new recombinant lipases and phospholipases that are “tailor-made” for given applications.

### 4.1. Host Systems for Heterologous Expression of Lipases and Phospholipases

The two general categories of expression systems are prokaryotic and eukaryotic. The prokaryotic systems include bacteria, and the eukaryotic systems include yeasts, filamentous fungi, insects and mammalian cells. Compared to insects and mammalian cells, bacteria, yeasts and filamentous fungi are generally easier to handle and represent economic heterologous platforms. This makes them more suitable for industrial applications, whereas insects and mammalian are largely used for the production of pharmaceuticals.

**Figure 6:**
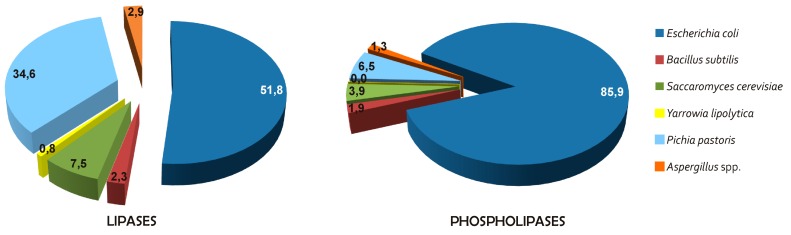
Percentage of use of the microbial hosts for heterologous expression of lipases and phospholipases. Data do not include enzymes autocloned and homologously expressed in the microorganism of origin.

The information concerning the microbial species that are most frequently used for heterologous expression of recombinant lipases and phospholipases are summarized below. To determine what might be the host of choice for heterologous expression of both lipases and phospholipases, searches were carried out in Scopus and the ISI Web of Knowledge. The results obtained revealed that *Escherichia coli* is the most widely used host for heterologous expression of both of these enzymes, whereas, among the yeasts and fungi, *Pichia pastoris* has the major role (Figure 6).

In particular, *E. coli* accounts for the greatest proportion of the recombinantly expressed phospholipases, whereas the lipases expressed in this host amount to little more than half of all of the recombinant lipases. This is because the heterologous expression of phospholipases has been mainly carried out for providing enzymes needed for structural and functional studies, for which *E. coli* remains the host of choice. On the contrary, as lipases represent the most important group of enzymes for industrial applications, a large number of recombinant lipases has been produced to exploit their potential uses as industrial biocatalysts, and thus the yeast and fungal systems are preferring, which for this purpouse represent more suitable hosts than bacteria.

#### 4.1.1. Prokaryotic Host Systems

Prokaryotic hosts are generally the most used for heterologous protein expression, as they are generally easier to handle, have short growth times, and reach high cell densities in inexpensive media. Also, their genetics are often well characterized and increasingly large numbers of cloning vectors and mutant host strains have become available. However, they have important drawbacks, most of which are related to the inability of bacterial hosts to perform post-translational modifications, such as correct protein folding, phoshorylation and glycosylation, and correct formation of disulfide bridges. Thus, when post-translational modifications are not required, a bacterial strain represents the best host for the expression of a recombinant protein.

Among the bacteria, the Gram-negative *E. coli* represents the most widely used host. Different *E. coli* strains and a wide range of molecular toolboxes (*i.e.*, vectors, promoters, tags) are commercially available to build up an expression system that is as suitable as possible for high-yield production of a correctly folded recombinant protein. In *E. coli*, recombinant proteins are generally expressed in the cytosolic space or can be targeted and displayed on the cell surface of the *E. coli* cells. The presentation of the recombinant protein at the surface of the *E. coli* cells is achieved by fusing it to a carrier protein [151]. Recombinant proteins can also be secreted into the extracellular space, but this process is complicated, and therefore it has not been widely used [152].

As shown in Figure 6, about 52% of all of the recombinant lipase systems use *E. coli* expression. These include almost exclusively the lipases from the bacterial strains that belong to the genera *Pseudomonas*, *Bacillus*, *Staphylococcus*, *Serratia* and *Burholderia*, and from metagenomic libraries of different environmental samples. A few lipases from the yeast genus *Candida*, which are mainly represented by CALB, have also been expressed in *E. coli.* Lipases expressed in this host have been used as biocatalysts almost exclusively in biodiesel production (see Section 5.1). As far as phospholipases are concerned, more than 86% of all of the recombinant enzymes are expressed in *E. coli*. Of these, half are PLA_2_s, followed (in decreasing order) by PLCs, PLDs, PLA_1_s and PLBs. Recombinant PLA_2_s mainly include sPLA_2_s derived from different animal venoms, human and animal tissues and fluids, and a few plant sources. All of the other recombinant phospholipases are mainly from bacterial and plant sources. Microbial phospholipases expressed in *E. coli* are used as biocatalysts mainly in oil degumming (see Section 5.3).

The second most used prokaryotic organism for recombinant protein expression after *E. coli* is the Gram-positive soil bacterium *B. subtilis*. Compared to *E. coli*, *B. subtilis* has the advantage that it can secrete the protein at high concentrations directly into the medium, by fusing it with N-terminal signal sequence. For the surface display of recombinant proteins on *B. subtilis*, the carrier proteins used can be anchored either in the cytoplasmic membrane or in the wall of the cell envelope. An interesting surface display system is based on the use of *B. subtilis* spores, whereby the proteins of the *B. subtilis* spore coat are used as carrier proteins [153].

As shown in Figure 6, *B. subtilis* is a bacterial host that has rarely been used for heterologous expression of either lipases or phospholipases. In both cases, the percentage of enzyme expressed in this host is around the 2%, and this exclusively includes bacterial lipases and phospholipases from *Bacillus*, *Pseudomonas* and *Staphylococcus* species.

#### 4.1.2. Eukaryotic Host Systems

Eukaryotic expression systems, such as yeasts and filamentous fungi, have several advantages when complex proteins have to be expressed. As unicellular organisms, yeasts retain the same advantages as prokaryotic organisms. In addition, as eukaryotic organisms, they have a sub-cellular organization that can carry out post-translational modifications. As yeasts are also food microorganisms, they are preferred to bacteria for use in food processing. 

The first yeast used for heterologous expression of recombinant proteins was the *S. cerevisiae*, which is also known as “baker”s yeast. This choice was based on the large amount of information about its genetics and physiology, and its classification as GRAS, which allows its wide use in food industry [150]. As a yeast, *S. cereviesiae* can display high-molecular-weight proteins on its cell surface [154].

Lipases expressed in *S. cerevisiae* represent 7.6% of all of the recombinant lipases (Figure 6). These mainly include lipases from yeast strains of the genus *Candida* and fungal strains of the genus *Rhizopus*. Among these, CALB and ROL are the lipases that are most frequently expressed in this host system, and a number of reports have dealt with their application as biocatalysts, particularly in biodiesel production (see Section 5.1). For phospholipases, *S. cerevisiae* accounts for only 3.9% of these recombinantly expressed enzymes (Figure 6), which mainly include PLA_2_s from different mammalian sources. Protein expression in *S. cerevisiae* suffers, however, from several limitations, such as plasmid instability, low production yields even in the presence of strong promoters, low secretory capacity, and hyperglycosylation of protein products [150]. For these reasons, other non-*Saccharomyces* yeasts have been considered as alternative expression systems.

Among these non-*Saccharomyces* yeasts, *P. pastoris* has been the most applied heterologous system for the production of commercially relevant recombinant proteins. Compared to *S. cerevisiae* it reaches higher expression levels and presents a reduced phenomenon of hyperglycosylation. However, the most important feature of *P. pastoris* is that it can use methanol as the sole source of carbon and energy [155]. Therefore, the switch from glucose to methanol medium induces high-level expression of the recombinant protein in this host. In *P. pastoris*, recombinant proteins can be expressed intracellularly, secreted into the medium, or displayed at the cell surface. As methylotrophic yeasts secrete low levels of their own proteins, secretion of a recombinant protein into the extracellular medium is preferred to the intracellular production as it represents a first step in the purification of a recombinant protein.

As shown in Figure 6, 34.6% of the recombinant lipases are expressed in *P. pastoris*. These mainly include yeast lipases from *Candida* sp., and fungal lipases from *Rhizopus* sp., *Yarrowia* sp., *Aspergillus* sp., *Rhizomucor* sp. and *Thermomyces* sp. Altogether, these lipases account for roughly 65% of all of the lipases that are recombinantly expressed in *P. pastoris*. Among these, the lipases that are most frequently expressed in this host are CALB, CRL, ROL and *Yarrowia lipolytica* lipase 2 (YlLip2). The remaining lipases that are expressed in *P. pastoris* include bacterial lipases, which mainly fall into the genera *Bacillus*, *Penicillium* and *Geotrichum*, as well as lipases from different mammalian sources. Some lipases expressed in this host system have been successfully applied to industrial biocatalysis, particularly in biodiesel production, food industry and synthesis of nutraceuticals (see Section 5.1 and Section 5.2). Phospholipases expressed in *P. pastoris* correspond to 6.5% of all of the recombinant phospholipases, and these include PLA_2_s from different sources, a few bacterial PLA_1_s, PLBs and PLCs, and a plant PLD. A few of the bacterial phospholipases that have been expressed in this host have been successfully used as biocatalysts, particularly in the degumming of vegetable oils (see Section 5.3).

Another non-*Saccharomyces* yeast that is used for heterologous expression of industrially relevant enzymes is the nonconventional yeast *Y. lipolytica*. Similar to *S. cerevisiae*, *Y. lipolytica* is considered to be nonpathogenic, and several processes based on *Y. lipolytica* have been classified as GRAS. Its particular characteristic is that it naturally secretes several enzymes into the culture medium, and it has a glycosylation pattern that is closer to that of mammalian cells than to that of *S. cerevisiae*.

As shown in Figure 6, *Y. lipolytica* is the least used host for the expression of recombinant lipases, as it accounts for only 0.8% of the total recombinantly expressed lipases. *Y. lipolytica* is mainly used for the homologous expression of its own YlLip2 gene, whereas, as far as heterologous lipases are concerned, it has been successfully used to express yeast and fungal lipases, such as CALB, ROL and TLL [156]. To date, *Y. lipolytica* has never been used for the expression of recombinant phospholipases.

Among the filamentous fungi, the GRAS status of the *Aspergillus* genus has made some *Aspergillus* species attractive as hosts for protein expression. These can grow on relatively inexpensive substrates, and can both produce and secrete very high amounts of recombinant proteins. As for expression in yeast, the foreign gene is placed in an expression vector behind a strong promoter and linked to a signal sequence, which directs the protein to the secretory pathway. High-level expression can be achived by using both constitutive and inducible promoters. Other strategies to increase the yield of protein production include integration of multiple copies of the foreign gene into the fungal genome, fusion of the foreign gene with a gene encoding a naturally well-secreted protein, and use of protease-deficient strains to prevent degradation of the secreted recombinant protein [157].

Among the *Aspergillus* spp., *A. oryzae* is most frequently used for recombinant expression of lipases and phospholipases. Recombinant lipases espressed in *Aspergillus* account for 2.3% of all of the heterologously expressed lipases (Figure 6), and these mainly include CALB, ROL, TLL and FHL. Interestingly, this host has been extensively used as a whole-cell biocatalyst; in particular, in its immobilized form, lipase-expressing *A. oryzae* has been successfully applied to biodiesel production (see Section 5.1). For phosholipases, *A. oryzae* is often used for homologous expression of its own PLA_1_ gene, whereas expression of heterologous phospholipases accounts for only 1.3% of all of the recombinant phospholipases (Figure 6), and this includes exclusively porcine PLA_2_s expressed in *A. niger*.

### 4.2. Genetic Strategies to Improve Properties of Lipases and Phospholipases

Despite the increasing number of new lipase and phospholipase variants that have been isolated from natural sources (see Section 3.1) and successfully applied as biocatalysts, native enzymes are not always suitable for certain industrial applications and their properties have therefore to be optimized. Genetic engineering nowadays represents the most efficient approach to generate a “tailor-made” enzyme for a given application. Two main strategies have been developed to design proteins that work better or under unusual conditions: rational design and directed evolution [12,158].

Protein engineering by rational design requires information about the three-dimensional structure of the protein, as well as about the relation between its structure and function. This information is needed to define the amino-acid residues to be mutated to modify any specific property of the protein. For this purpose, a number of databases have been developed to give information on protein sequences, on catalytic residues, and on annotated mutants. For instance, the LED was developed to integrate sequence information for lipases and related proteins deposited in other public resources (e.g., GenBank, ExPDB, DSSP) and to allow the identification of functionally relevant amino-acid residues [35]. Once the amino-acid residue to be mutated has been identified, this is targeted for site-directed mutagenesis (SDM), which allows its substitution with another amino-acid residue [159]. Currently, a variety of PCR-based commercial kits are available (e.g., QuickChange^®^ by Agilent Technologies, GenArt^®^ by Life Technologies, Q5^®^ by NEB), which allow the deliberate incorporation of an amino-acid residue into a specific position. Site saturation mutagenesis (SSM) extends the utility of SDM, as it allows the substitution of a given amino-acid residue by any one of the other 19 amino-acid residues, thus generating a small library of mutants that contains all of the possible mutations at one pre-determined target position in a gene sequence [160].

Contrarily to rational design, directed evolution does not require information about the relationship between protein structure and function and can be applied fruitfully in the absence of structural data. This approach is based on the random mutagenesis of the target gene leading to the generation of a mutant library, which is then screened to identify the desired variant [12]. Two different approaches can be used to generate the mutant library: a non-recombining evolution and a recombining evolution. In the non-recombining evolution, a target gene is subjected to point mutations that are induced by error-prone polymerase chain reaction (epPCR), chemical mutagenesis, or UV irradiation. In the recombining evolution several parental genes are shuffled and reconstructed to generate a pool of recombinant chimera. The parental genes can comprise either naturally occurring homologs of a gene family, or selected mutants of a single gene generated by other mutagenesis methods [12].

Recently, methods that combine rational design and directed evolution have been developed, and these are defined as semi-rational design or focused directed (designed) evolution. These methods are advantageous when information about the relation between the protein structure and function suggest that a certain region is linked to a desired property, although without any idea as to which specific amino-acid substitution would be best. Hence, the amino-acid residues in interesting regions are randomly mutated, one by one or in combination, which can result in synergistic effects that might have been missed in single-site specific mutagenesis. This approach leads to the generation of smaller libraries compared to directed evolution, thus reducing the screening efforts needed [13]. An example of semi-rational design is the combinatorial active site saturation test (CASTing), in which a small set of amino acids in the vicinity of the active site is chosen and mutated randomly, followed by screening for the best hits [161]. Iterative saturation mutagenesis introduces an evolutionary factor into the CASTing approach, as this involves the use of iterative cycles of saturation mutagenesis at a rationally chosen site until the desired degree of improvement is reached [162].

After mutation, the gene variants are cloned, transformed in the host system, and expressed. Then, high-throughput screening is required to identify the best desired variant. These methods can be applied *in vivo*, so directly on colonies growing on agar plates, or *in vitro*, and thus after picking colonies and growing individual clones in liquid culture. The *in vivo* selection methods have the advantage that they allow the screening of high numbers of colonies simultaneously (roughly 10^4^ per day), although they can lack sensitivity. In the *in vivo* selection, colorimetric methods can be applied to agar plate colony screening. In these methods, colonies that show the desired color are selected as positive. Examples of *in vivo* selection include the identification of lipolytic clones through formation of haloes around the positive colonies in tributyrin agar plates. Alternatively, a triolein agar plate with Victoria Blue or Rhodamine B as an indicator can be used [163]. For phospholipases, soybean lecithin is used to identify colonies with PLA_1_, PLA_2_ or PLC activity. Also in this case, a halo is formed around the colony, whereby PLC produces a turbid zone, and PLAs produce a clear zone [164]. A colorimetric method has been reported for the detection of PLD activity that is based on the use of phosphatidyl-2-naphthol as the substrate and the PLD-catalyzed generation of the colored water-insoluble azo dye [165].

*In vitro* cell-free selection relies on determination of the enzymatic activity through colorimetric or fluorimetric measurements. This type of selection is more time consuming compared to *in vivo* selection, but it has the advantage that it can provide more information about the enzymatic activity. Lipase activity can be determined by the interaction of rhodamine B with the FAs released during the enzymatic hydrolysis of triglycerides, or by the hydrolysis of *p*-nitrophenyl esters followed by the appearance of the yellow colored *p*-nitrophenol, or by the dimercaptopropanol tributyrate method, in which -SH groups formed from lipase cleavage of dimercaptopropanol tributyrate react with 5,5′-dithiobis(2-nitrobenzoic acid) to form a yellow-colored product. For phospholipases, thio- or fluorescence-labeled phospholipds are used to assay for PLA_1_, PLA_2_ or PLC activity [165]. PLA_2_ activity can also be monitored using a lipoxygenase-coupled spectrophotometric assay with 1-palmitoyl-2-linoleoyl-*sn*-glycero-3-PC as the substrate. Here, the generation of free linoleate due to PLA_2_ hydrolysis is monitored by following its conversion into the conjugated diene hydroperoxide, which is catalyzed by the coupled lipoxygenase reaction [166].

#### 4.2.1. Engineering for Improvement in Activity

Rationally designed modifications near or at the active site have been successfully used to improve the enzymatic activities of both lipases and phospholipases. For instance, the SDM approach has been applied to increase the activity of CALB, by changing the edges of the four helices that surround the active site into flexible amino acids [167]. Two mutants were identified, V139E and I255E, that showed specific activities that were 5.4- and 3.5-fold higher, respectively, compared to those of the wild-type (WT) CALB. The same approach was used by Kolling and coworkers [168] to enhance the activity of a *Staphylococcus xylosus* lipase. In this study, the V309 next to the catalytic A306 was substituted with aspartate and lysine, and both of these mutants showed specific activities that were >2-fold higher compared to the WT enzyme. Also, a SSM approach was used to mutate the G152 and S154 residues in lid1 of *Pseudomonas* sp. acidic lipase Lip I.3. Here, the two mutants showed 5- and 11-fold increased activity, thus suggesting that modifications to the structure of lid1 can promote improvements in the catalytic efficiency [169].

Successful results for the improvement of lipase activity have also been achieved using directed evolution strategies. The epPCR approach was used to improve the activity of *Rhizopus chinensis* lipase (RCL) [170]. Two mutants were obtained, 1–11 and 2–28, that showed activities that were 2- and 4-fold higher, respectively, than that of the WT enzyme. Sequence and structural analysis of the mutant 2–28 revealed the presence of four amino-acid substitutions that were located on the protein surface: A129S, K161R, A230T and K322R. Among these, the K322R substitution that was located in a loop near the catalytic center of the lipase pulled the loop in the opposite direction of the substrate channel, thus making it easier for the substrate to enter the lipase catalytic domain. An approach based on epPCR and DNA shuffling methods was carried out to improve the activity of a *Proteus vulgaris* lipase [171]. A single triple mutant was obtained that showed an activity that was 3.5-fold higher than that of the WT enzyme. Of the three mutations here, V102L was located near the catalytic active site, and it was the only one found to be critical for the lipase activity. DNA shuffling has also been applied to three homologous lipases B from *C. antarctica* ATCC 32657, *Hyphozyma sp*. CBS 648.91 and *Crytococcus tsukubaensis* ATCC 24555, to generate a diverse gene library [172]. The screening identified a chimeric lipase B that had a 20-fold higher activity than lipase B from *C. antarctica* ATCC 32657, and a 13-fold higher activity than the most active parent, which was derived from *C. tsukubaensis* ATCC 24555.

With regard to phospholipases, a PLD exotoxin from *Corynebacterium pseudotuberculosis*, which is the major virulence factor of this microorganism, was recently mutagenised using SDM to substitute a histidine residue in the active site with a tyrosine residue. This mutation resulted in a loss of the hemolytic activity of the PLD, although the immunogenic and antigenic activities of the protein were maintained, thus suggesting its use in the formulation of recombinant PLD vaccines [173]. SDM was also applied by Ogino and coworkers [174] to mutate the catalytic activity of a PLD from *Streptoverticillium cinnamomeum*. The mutation consisted of the substitution of a glycine residue in the GG conserved motif located downstream from each HDK motif with a serine residue. The mutants obtained showed transphosphatidylation activity that was from 9- to 27-fold higher than that of WT PLD.

#### 4.2.2. Engineering for Modification of Chemo-Specificity and Enantio-Selectivity

Substrate specificity largely depends on the interactions between the substrate and the substrate-binding site. So mutations can be rationally designed on the basis of structural models, to change the shape, size and/or hydrophobicity of the substrate-binding site, and thus to improve the chemo-specificity or enantio-selectivity of a given enzyme.

Recently, two highly *trans*-selective variants, T221H and I301H, of *C. antarctica* lipase A (CALA) that efficiently remove *trans*-FAs from partially hydrogenated vegetable oils were developed using SDM of the FA binding tunnel [175]. These variants showed a straight and narrow FA binding tunnel that only allowed access to FAs that can adopt linear geometries (e.g., saturated and *trans*-FAs), and hindered access to FAs that cannot (e.g., *cis*-FAs). The same approach was used by Schmitt and coworkers [176] to mutate CRL at amino-acid residues positioned in different locations inside the tunnel (*i.e.*, P246F, L413F, L410W, L410F/S300E, L410F/S365L). The results that they obtained demonstrated that the activity for FAs sharply decreased as they became long enough to reach the mutated site.

In addition to the substrate-binding site, there is evidence that other protein regions, such as the lid, might have a role in lipase specificity. Indeed, when a disulfide bridge was introduced between N84C and G266C in the lid of RCL using SDM, a decrease in the affinity for long-chain FAs was observed [177]. This appears ascribable to hindrance generated by the disulfide bond introduced at the end of the binding domain, which might interfere with the binding of the long-chain FAs. Also, when the lid of *Pseudomonas fragi* lipase was specifically targeted using SDM, two substitutions to lid positions 137 and 138 were found to increase the activity toward C8 FAs [178].

For the improvement in enantio-selectivity, a SDM approach has been used to invert the enantio-selectivity of the YlLip2 lipase, an enzyme that is characterized by a low *S*-enantio-selectivity during the resolution of 2-bromophenyl acetic acid esters. Cambon and coworkers [179] mutated two positions that are known to have key roles for enantiomer discrimination, amino acids 97 and 232, and they obtained a double mutant, D97A-V232F, that was fully specific for the *R*-enantiomer. This position in YlLip2 was also mutated by Bordes and coworkers [180], and their variant, V232A, showed enantio-selectivity enhanced by one order of magnitude, compared to the WT enzyme, whereas their variant V232L showed a selectivity inversion. SDM has also been applied to mutate *C. rugosa* Lip1 and Lip4 in position 296, which is known to have a role in the *S*-enantiomer or *R*-enantiomer specificity of the YlLip2 [181]. Screening of the variants for their enantio-selectivity toward 2-bromo phenyl acetic acid octyl esters revealed that the bulkier the amino acid at position 296, the greater the specificity toward the *S*-enantiomer. A highly combinatorial structure-based protein engineering method was developed to reshape the substrate binding pocket of CALA, to enhance the enantio-selectivity of this enzyme toward the 2-(4-isobutylphenyl)propanoate ester (ibuprofen ester) [182]. Two mutants, SV_1_CV_2_ and SV_1_CAV_2_ that carried four and five mutations, respectively, were identified, which were characterized by an *S*-enantio-preference toward the ibuprofen ester, which was a lot higher compared to WT CALA.

For phospholipases, the SDM approach has been successfully applied to engineer their substrate-specificity. Notably, this was applied to change the glutamate 46 to leucine in porcine pancreatic PLA_2_, as the removal of a negative charge in the hydrophilic region of the phospholipid binding site was believed to influence the head-group selectivity of this enzyme [183]. The E46L mutant showed an activity toward 1,2-dilauroyl-*sn*-glycero-3-phosphate, which was three-fold higher than that observed for 1,2-dilauroyl-*sn*-glycero-3-phosphocholine. Moreover, contrary to the native PLA_2_, the mutant PLA_2_ was not inhibited by the products released from the hydrolysis of 1,2-dilauroyl-*sn*-glycero-3-phosphate. Also, SDM was used to modify the substrate specificity of a PLC from *B. cereus* by mutation of the E4 residue [184], which has been proposed as a potential candidate for the general base in the hydrolysis reaction, and has been shown to be critical for the enzyme specificity for phosphodiester over PA substrates. The substitution of the glutamate 4 residue with an alanine resulted in significant increase in PA hydrolysis, and decrease in PC hydrolysis. Moreover, the E4A mutant did not show interfacial activation, which suggested that the highly flexible loop in which the glutamate residue was located and that flanked the entrance to the active site might constitute an interfacial binding-recognition site.

#### 4.2.3. Engineering for Improvement in Stability in Organic Solvents

The stability of an enzyme in organic solvents is mainly influenced by surface properties and surface charge distribution of the protein. So, to enhance the stability of CALB in hydrophylic organic solvents, a SDM approach has been developed by Park and coworkers [185] to induce single mutations in random coils or loops at the enzyme surface that improved the network of hydrogen bonds. Three mutants were identified, N97Q, N264Q and D265E, which showed greater stability and longer half-life in the presence of 80% methanol. A SDM approach aimed at replacing the polar amino acids with hydrophobic residues at the protein surface has also been applied to a lipase from *Pseudomonas* sp. [186]. The two mutants obtained, N219L and N219I, showed stabilities that were significantly increased in the presence of different organic solvents, compared to the WT enzyme. Instead, SSM was used to engineer a *B. subtilis* lipase [187]. With this approach, all 91 amino acids of the surface loops of the enzyme were mutated, and among these, six mutations were identified that contributed to higher activities in dimethyl sulfoxide (DMSO). The combination of the six mutations into one gene generated a new mutant, 6SR, which was characterized by a more polar surface, and which showed a catalytic turnover in 60% DMSO that was 8-fold higher compared to the WT enzyme.

Improvements in organic solvent stability can also be achieved by increased packing of the hydrophobic core in the lipase. For instance, various mutants of a lipase from *P. aeruginosa* LST-03 have been prepared using SDM [188]. Among these, the S155L, G157R, S164K, S194R, and D209N mutants were identified, which resulted in the formation of a salt bridge and hydrogen bonds, as well as in the improvement of the packing of the hydrophobic core. All of these contributed to the increase in protein stability in the presence of various organic solvents.

Among phospholipases, a directed evolution approach has been used to enhance the stability in organic solvents of a PLA_1_ from *Serratia* sp. MK1 [189]. In particular, a two-step evolutionary engineering approach was applied, which consisted of the preparation of a mutant library by epPCR screened by exposure to 30% DMSO, followed by DNA shuffling of the positive clones, with final screening in 50% DMSO. Three mutants, SA8, SA17 and SA20, were identified that had a half-life in 50% DMSO that was 4-fold higher compared to WT PLA_1_. They also showed superior stability in most of the organic solvents. The three mutants had five of their eight amino-acid substitutions over only a 30-residue portion (less than 10% of the whole protein), thus suggesting that the corresponding region of PLA_1_ might be particularly important for stability and activity in DMSO.

#### 4.2.4. Engineering for Improvement in Stability at High and Low Temperatures

The factors generally responsible for modulation of the stability at high and low temperatures are those related to the flexibility/rigidity of the enzyme structure. Of note, a more rigid structure is generally associated with a higher stability at high temperatures, whereas flexibility has long been hypothesized to be an important requirement in cold adaptation.

SDM was carried out on CALB to introduce a new disulfide bridge [126]. The A162C-K308C mutant was indentified, which showed an increased rigidity and greatly improved thermostability compared to that of WT CALB. The same SDM approach was used to achieve an increase in the thermostability of RML [190]. Two mutations were created, P96C and K106C, to introduce a new disulfide bridge, and the variant obtained, mRML66, showed a half-life at 60 °C that was approximately 5-fold higher than that of WT RML. When displayed on *P. pastoris* cell surface, mRML66 had a residual activity after incubation at 60 °C for 5 h, which was significantly higher than the native enzyme. An epPCR approach was used to increase the thermostability of YlLip2 [191]. A thermostable variant was identified that had only one mutation, as C244A. SSM at position 244 demonstrated that the free cysteine 244 in the WT enzyme induced rapid intermolecular disulphide bridge interchanges that triggered aggregation and reduced thermostability. Recently, a combinatorial protein engineering approach was used to improve the thermostability of a lipase (LipR1) of metagenomic origin [192]. The variant LipR5 was generated after combination of two thermostabilizing mutations: R214C and N355K. This variant showed a thermostability at 60 °C that was nearly 168-fold higher compared to LipR1. Circular dichroism and fluorescence studies have suggested that the protein structure had become more rigid and was stable to denaturation.

An example of lipase engineering that was aimed at evolving an enzyme active at low temperatures is seen in the study carried out recently on the *B. subtilis* lipase by Kumar and coworkers [193]. As loops are known to be flexible regions in the protein structure, a mutant library generated by SSM of 88 amino-acid positions in the loop regions of the WT lipase was screened for cold-active mutants. Five mutations were identified which enhanced the enzyme activity at low temperatures; a recombinant of these five mutations, named as 5CR, showed 7-fold higher catalytic efficiency than the WT lipase at 10 °C. Interestingly, four out of the five single mutations were glycine substitutions in the loop lying close to the active site. As glycine residues are known to relieve mobility restrictions in a peptide backbone, it is feasible that these might have contributed to the flexibility of the active site, and thus might facilitate activity at low temperatures.

Among the phospholipases, the thermal stability of a PLA_1_ from *Serratia* sp. MK1 was improved by sequential rounds of epPCR [194]. This approach allowed the identification of two mutants, TA3 and TA13, which differed from WT PLA_1_ in terms of a tighter packing of the hydrophobic core, and of the presence of additional hydrogen bonds, respectively. Another example is a study carried out by Negishi and coworkers [195] that was aimed at identifying the relationship between the structure and thermal stability of *Streptomyces* PLDs. The authors applied the *in vivo* DNA shuffling method to construct a chimeral-gene library between the thermostable PLD from *Streptomyces septatus* TH-2 (TH-2PLD) and the thermolabile PLD from *Streptomyces halstedii* K1 (K1PLD). The results obtained strongly suggest that G188 is the key amino-acid residue that determines the thermostability of this enzyme. Indeed, the substitution of this glycine residue with a hydrophobic amino acid, such as phenylalanine, valine or tryptophan, resulted in increased thermostability, which was probably due to tighter packing of the PLD protein.

#### 4.2.5. Engineering for Improvement in pH Stability

There have been very few attempts to engineer lipases and phospholipases with the aim of improving the tolerance of these enzymes to extreme pH, apparently because stabilization of the protein tertiary structure does not necessarily lead to an increase in pH tolerance.

Among lipases, epPCR combined with SDM was used to improve the performances of human pancreatic lipase at an acidic pH [196]. A lipase variant was identified that showed a 1.5-fold increased activity at low pH compared to the WT enzyme. Also, an increased pH stability was observed for four mutants, L132A, L132I, G450S and G450A, which were obtained through SSM on *C. rugosa* Lip2 [197], as well as in the BpLe-1369 mutant that was obtained through two cycles of epPCR on the *B. pumilus* lipase YZ02 [198].

For phospholipases, SDM has been applied to tailor the pH dependence of human nonpancreatic sPLA_2_, which was characterized by high activity under alkaline conditions, but low activity in the neutral pH range [199]. Three surface-charge replacements, T42E, R100E, and E89K, generated mutants that showed an activity at pH 7 that was from 2.3- to 2.8-fold higher compared to the activity of WT PLA_2_. Both the positive-to-negative and negative-to-positive mutations lowered the optimum pH of this sPLA_2_, thus indicating that the pH profile of this enzyme depends on a delicate balance of charged residues on its surface.

## 5. Industrial Applications of Recombinant Lipases and Phospholipases

Over the last two decades, there has been a tremendous increase in the significance of biotechnological applications of lipases and phospholipases. The use of these enzymes in industrial processes has grown hand-in-hand with the ability to clone and express their genes in heterologous hosts so as to obtain commercially relevant amounts of these enzymes. In addition, the industrial use of both lipases and phospholipases is increasing due to the optimization of their properties through protein engineering.

Lipases and phospholipases produced by the heterologous host can be used as free enzymes after purification from the producing organism, or they can be used while still contained within the producing organism. Both free enzymes and whole-cell systems can be immobilized on suitable matrices or supports. However, for some applications of immobilized lipases, the relatively high cost associated with the usage of an expensive support material becomes prohibitive for industrial uses. Therefore, further development of a less expensive immobilization process technology is necessary for the use within low margin applications, such as oils and fats industry, as compared to high margin applications, such as enantio-selective synthesis of pharmaceutical intermediates [200]. On the other side, immobilization has the advantage to increase the chance of enzyme/whole cell re-usability, and eases the separation of the biocatalyst from the products. In some cases, this also leads to an improvement in the activity, specificity and stability of the enzyme, thereby allowing its application to industrial processes that are carried out under relatively harsh conditions [201]. All these advantages may allow reducing the cost in the use of an immobilized biocatalyst; this, together with a low cost of recombinant enzyme production, can render immobilisation attractive for a wider range of process applications. In recent years, yeast cells that display the recombinant enzyme on their cell surface have been emerging as whole-cell systems that are more suitable for applications in biocatalysis. Indeed, enzyme-displaying yeast cells have the typical advantage of whole-cell systems, that is the avoidance of the need for enzyme purification from the producing microorganism, and also present the advantage to be directly used as biocatalysts without the additional step of immobilization, as displaying the enzyme on the cell surface of the host cell represents a type of self-immobilization.

An overview is given below relating to the efforts that have been made by various research groups to develop recombinant lipases and phospholipases that meet the specific needs of these enzymes in industrial processes. The picture that emerges from the findings is that despite the high potential of lipases and phospholipases, their large-scale application is to date mainly limited to the dairy, baking and detergent industries, with more than 50 enzymes fully developed and commercialized in the past 25 years. As a consequence, current research efforts in these application areas are limited. On the contrary, the large-scale application of recombinant lipases and phospholipases to other industrial processes, such as biodiesel production, oil degumming, and synthesis of flavor compounds and nutraceuticals, is still under development, and extensive research is in progress to develop full heterologous expression systems and design tailor-made enzymes that allow the drawbacks that limit their use in such relevant industrial processes to be overcome.

### 5.1. The Biodiesel Industry

Biodiesels can be produced from non-edible or low-value feedstocks, such as animal fats, vegetable and microalgal oils, waste products of vegetable oil refineries and animal rendering, and used cooking oils. They represent one of the most promising sustainable alternatives to decrease dependence on traditional fossil fuel [202]. Biodiesels are a mixture of FAAEs that are usually produced by esterification of FFAs or transesterification of TAGs. Currently, the world supply of biodiesels comes almost exclusively from base-catalyzed transesterification of TAGs with short-chain alcohols, such as methanol and ethanol. However, this chemical process has some drawbacks, as it requires downstream purification to neutralize the catalyst and to separate the biodiesel and glycerol. This latter process is complicated by the large amount of soaps generated during the reaction. A downstream process is also needed for the treatment of the large quantities of wastewater produced from the process. Moreover, alkali-catalyzed transesterification is very sensitive with respect to the feedstock purity, as it needs raw materials that are essentially free of phosphatides, FFAs and water [203]. For all of these reasons, this alkali-catalyzed transesterification represents an expensive and nonecologically friendly process.

Lipase-catalyzed transesterification is becoming more and more attractive, as this has several advantages compared to chemical catalysis. Indeed, lipases can catalyze not only TAG transesterification reactions, but also FFA esterification with an alcohol, thus making it possible to produce biodiesel from used oils or fats with high FFA levels. Moreover, lipases produce FAAEs without forming soaps, thus allowing easier glycerol recovery and fuel purification; they are also highly selective, require mild reaction conditions, and produce fewer side products and low amounts of wastewater. Among the commercially available lipases, the most widely used in biodiesel production are free lipases from *P. fluorescens* (Lipase AK, Amano), *B. cepacia* (Lipase PS, Amano), and *T. lanuginosus* (Lipase LA201 and Lipopan 50BG, Novozymes), and immobilized lipases from *T. lanuginosus* (Lipozyme TL IM, Novozymes) and *R. miehei* (Lipozyme RM IM, Novozymes) [88]. However, the high cost of lipase production is the major obstacle to the use of this enzyme for industrial-scale biodiesel production. As a consequence, biodiesel derived from chemical transformations still dominates the current global market, whereas the application of lipases on an industrial scale has been achieved only in China, where the first plant for biodiesel production using lipases came into operation in 2006. Recently, the successful application of lipases to biodiesel production was also demonstrated by a pilot-scale plant that came into operation in Brazil, which is considered a leader in the biodiesel economy [204].

Among recombinant lipases, CALB expressed in *A. niger* and immobilized onto an acrylic macroporous resin, that is commercially known as Novozym 435 (Novozymes), is the lipase that is most widely used for the production of biodiesel [88]. This recombinant lipase has been successfully used in conventional biodiesel production, as well as in biodiesel production using isopropanolysis of soybean oil, simultaneous synthesis of biodiesel and glycerol carbonate from corn oil using dimethyl carbonate as the acyl acceptor, and production of so-called “Ecodiesel”, which contains a mixture of MAGs and FAAEs that are obtained through partial ethanolysis of TAGs in sunflower oil [205].

In addition to Novozym 435, several other recombinant lipases have been produced using different host organisms, and these have been successfully applied to biodiesel production (Table 4). Lipases expressed in the *E. coli* host system have been used almost exclusively in their purified form, as free or immobilized enzymes. Among these, the new variant of *Proteus mirabilis* lipase is worth mention, which was developed by means of a directed evolution approach by Korman and coworkers [206], and is called Dieselzyme 4. This enzyme was found to be able to convert canola oil into biodiesel with high yields (76% and 100% in the first and second cycle, respectively). Moreover, compared to the WT enzyme, the Dieselzyme 4 variant presented an improved methanol tolerance that was even more evident when the enzyme was immobilized and reused. Indeed, while the native *P. mirabilis* lipase lost nearly all of its activity in the second cycle of transesterification, the new variant completely retained its activity even after a fourth cycle of transesterification.

Only in a few cases have *E. coli*-expressing lipases been used in biodiesel production as a whole-cell biocatalyst (Table 4). *E. coli* expressing lipK107, which is a lipase produced from a soil-bacteria strain of *Proteus* sp., represents the first example of an *E. coli*-expressing lipase applied to biodiesel production as a whole-cell biocatalyst [207]. This system produced yields of about 100% at a temperature of 15 °C, which is the lowest temperature among all of the known catalysts used in biodiesel production. An *E. coli* whole-cell system that co-expresses CALB and TLL has also been used to produce biodiesel from waste grease, and in this case, FFA esterification and TAG transesterification were simultaneously achieved with a yield of 87% and 95% using wet and dry cells, respectively [208].

Recombinant lipases produced by the yeasts *S. cerevisiae* and *P. pastoris* have been used as crude supernatants, purified enzymes, and whole-cell systems (Table 4). An interesting application of crude lipases to directly catalyze biodiesel production is that reported by Guan and coworkers [209]. In this study, the 1,3-specific RML and the MAG- and DAG-specific lipase from *P. cyclopium* (MDL) were separately expressed in *P. pastoris*, and the supernatant of the two fermentation broths was used to catalyze the transesterification of soybean oil with methanol. The results obtained demonstrated that the transesterification yield of RML assisted by MDL was higher than that of RML alone (95% *versus* 68.5%, respectively), thus suggesting that MDL addition is helpful to assist RML in the hydrolysis of both MAGs and DAGs.

Recombinant lipases purified from their expressing yeast systems have been used as free or immobilized enzymes, and these include CALB, ROL, RML and lipZ03, where this last is a lipase that is encoded by a gene isolated from an oil-contaminated soil-derived metagenomic library. For CALB, different synthetic variants have been generated using automated PCR, and these have been tested for biodiesel production from corn and soybean oil. In particular, Hughes and coworkers [210] fused the CALB gene with the sequence encoding the Lyt-1 peptide, to facilitate the movement of the enzyme out of the *S. cerevisiae* cell. As expected, the supernatant of the yeast cells expressing this CALB Lyt-1 fusion protein had a specific activity almost 4-fold higher compared to the supernatant of the yeast cells expressing CALB alone. Both the purified CALB Lyt-1 immobilized on a resin support and the intact yeast cells expressing CALB Lyt-1 had specific activities greater than Novozym 435 (80 and 160 µg FAEEs/mg lipase *versus* 40 µg FAEEs/mg lipase, respectively). With the same approach, Hughes and coworkers [211] obtained a truncated variant of CALB that lacked the initial 34 amino-acid residues. This truncated CALB, once expressed and purified from *P. pastoris*, was immobilized on a resin support and tested for biodiesel production from corn and soybean oils, and it was shown to have a specific activity 36% and 50% higher, respectively, than Novozym 435. High yields (>90%) have also been obtained using recombinant RML [212,213] and ROL [214] expressed in *P. pastoris* and used in their free and immobilized forms, respectively. This was achieved by inducing high expression levels; in the first case, with the introduction of two copies of the *RML* gene and, in the second case, with the replacement of the prosequence of ROL with that of RCL.

Among the lipase-producing yeasts that have been used as whole-cell systems, of note, there is the dual biocatalytic system that couples lipase production and *in-situ* biodiesel synthesis [215]. This system was based on a recombinant *P. pastoris* strain transformed with a heterologous lipase-encoding gene from *T. lanuginosus*, which can simultaneously grow, overexpress the lipase, and efficiently catalyze biodiesel production from waste cooking oils with an 87% yield. However, among the yeast whole-cell systems, the most promising biocatalyst for the production of biodiesel on an industrial scale has been with those systems that have the lipase on the cell surface. Cell-surface-displayed lipases were used for the first time in biodiesel production by Matsumoto and coworkers [216], who displayed ROL on the cell surface of *S. cerevisiae* cells. This system successfully catalyzed the methanolysis reaction with yields higher than 78% after 72 h of reaction. A RML-displaying *P. pastoris* whole-cell biocatalyst has also been developed and used for biodiesel production from soybean oil [217]. This system reached a yield of 83% after 72 h, which was higher than that previously obtained using ROL-displaying *S. cereviasiae*. Moreover, the RML displayed on *P. pastoris* cells had good storage stability and maintained more than 80% of its original activity even after 10 cycles of transesterification. Recently, to improve biodiesel production, RML displayed on *P. pastoris* cells has been used in combination with CALB, which was also on the *P. pastoris* cells [218]. When used for transesterification of different feedstocks, these two displayed lipases reached yields that were higher than those for the RML-displaying *P. pastoris* cells alone, and the time to obtain maximum yield was shortened from 72 to 12 h. Compared to the lipase-displaying systems previously used, this new combined system showed an improved stability, as its activity remained almost unchanged after storage for 1 year at room temperature, and the yield remained >85% after 20 repeated batch cycles.

The lipase-expressing *A. oryzae* has been used in biodiesel production exclusively as a whole-cell biocatalyst immobilized on biomass support particles. For the first time Hama and coworkers [219] applied immobilized *A. oryzae* cells transformed with the gene that encodes the FHL in the enzymatic production of biodiesel. Since then, there have been several reports on the use of immobilized *A. oryzae* cells that express FHL in biodiesel production, using oils from different sources [220,221,222]. To improve the efficiency of the expression system, different strategies have been used, such as multiple copy insertion of the expression cassette [220] and the use of the improved enolase promoter (P-enoA124) together with the 5′-UTR of a heat-shock protein [221]. Thus, high yields (90%–100%) have been achieved in biodiesel production, and these systems have also shown to maintain high lipase activity upon reuse. Adachi and coworkers [223,224] also immobilized *A. oryzae* cells that expressed CALB and a thermostable and solvent-tolerant lipase from *Geobacillus thermocatenulatus*; this latter system reached nearly 100% yield at high temperatures (40–50 °C) and high methanol concentrations.

**Table 4:** Biodiesel production by recombinant lipases.

Host Strain	Donor Species	Lipase Name	Feedstock	Alcohol	Alcohol:Oil Ratio	Lipase Form	Temp. (°C)	Yield (%)	Refs.
*Escherichia coli*	*Pseudomonas aeruginosa*	Lipd25A	*Jatropha* seed oil	Methanol	3:1 (*n*:*n*)	Purified and immobilized	33	75	[225]
	*Bacillus amyloliquefaciens*	LipBA	Olive oil	Methanol	3:1 (*n*:*n*)	Purified and immobilized	35	~100	[226]
	*Bacillus subtilis*	Lipase I4-2	Soybean oil	Methanol	3:1 (*n*:*n*)	Purified	37	98	[227]
	*Proteus mirabilis*	Dieselzyme 4	Canola oil	Methanol	5.1 (*n*:*n*)	Purified and immobilized	25	76, 100	[206]
	*Staphylococcus haemolyticus*	Lipase L62	Olive oil	Methanol	6:1 (*n*:*n*)	Purified and immobilized	30	90	[228]
	*Candida antarctica/Thermomyces lanuginosus*	CALB/TLL	Waste grease	Methanol	1:1 (*n*:*n*)	Whole-cell (wet and dry)	30	87, 95	[208]
	*Bacillus thermocatenulatus*	BTL2	Soybean oil	Methanol	2 mL in 100 g	Purified	45	94.8	[229]
	*Proteus vulgaris*	Lipase K80	Plant and waste oil	Methanol	3:1 (*n*:*n*)	Purified and immobilized	30	78-100	[230]
	*Proteus* sp. (soil sample)	LipK107	Olive oil	Methanol	5:1 (*n*:*n*)	Whole-cell	15	~100	[207]
	*Pseudomonas fluorescens*	LipB68	Soybean oil	Methanol	3:1 (*n*:*n*)	Purified and immobilized	20	92	[231]
*Saccharomyces cerevisiae*	*Candida* sp.	Lip2	*S. cerevisiae* lipids	Ethanol	4% (*v*/*v*)	Whole-cell	30	11.4 mg/g cell dry weight	[232]
	*Candida antarctica*	truncated CALB	Corn and soybean oil	Ethanol Butanol	5:1 (*n*:*n*)	Purified and immobilized	37	894–1394 µg/mg of lipase	[211]
	*Candida antarctica*	CALB Lyt-1	Corn and soybean oil	Ethanol	15:1 (*v*:*v*)	Whole-cell; Purified and immobilized	50	160 µg/mg of lipase, 80 µg/mg of lipase	[210]
	*Rhizopus oryzae*	ROL	Soybean oil	Methanol	1:1 (*n*:*n*)	Whole-cell surface-displayed	37	>78	[216]
	*Rhizopus oryzae*	ROL	Soybean oil	Methanol	1:1 (*n*:*n*)	Whole-cell	37	71	[233]
*Pichia pastoris*	*Rhizomucor miehei*	GH2	Microalgae oil	Methanol	30:0.1 (µL:g)	Purified	30	>90%	[212,213]
	*Thermomyces lanuginosus*	Tll	Waste cooking oil	Methanol	3:1–9:1 (*n*:*n*)	Whole-cell	30	50%–87%	[215]
	*Thermomyces lanuginosus*	Tll	Waste cooking oil	Methanol	4:1 (*n*:*n*)	Whole-cell	40	82%	[234]
	Oil contaminated soil-derived metagenomic library	lipZ01	Olive oil	Methanol	3:1 (*n*:*n*)	Crude supernatant	37	~92%	[235]
	*Rhizopus oryzae/Rhizopus chinensis*	Chimera lipase	Tung oil	Methanol	3.88:1 (*n*:*n*)	Purified and immobilized	40	92%	[214]
	*Candida antarctica/Rhizomucor miehei*	CALB/RML	Different feedstocks	Methanol	3:1–7:1 (*n*:*n*)	Whole-cell surface-displayed	50	>90%	[218]
	Oil contaminated soil-derived metagenomic library	LipZ03	Olive oil	Methanol	3:1 (*n*:*n*)	Purified	37	74%	[236]
	*Rhizomucor miehei*	RML	Soybean oil	Methanol	3:1–9:1 (*n*:*n*)	Whole-cell surface-displayed	55	83%	[217]
	*Rhizopus oryzae*	ROL	Soybean oil, *Jatropha* *curcas* and *Pistacia chinensis* seed oil	Methanol	0.35:5 (g:g)	Crude supernatant	35	92%–95%	[237]
	*Rhizomucor miehei/Penicillium cyclopium*	RML/MDL	Soybean oil	Methanol	4:1 (*n*:*n*)	Crude supenatant	25	>95%	[209]
*Aspergillus oryzae*	*Candida antarctica*	CALB	Soybean and palm oil	Methanol	1.74:200 (g:g)	Whole-cell immobilized	30, 50	90%	[223]
	*Geobacillus thermocatenulatus*	BTL2	Palm oil	Methanol	0.37:9.63 (g:g)	Whole-cell immobilized	40-50	~100%	[224]
	*Fusarium heterosporum*	FHL	Shirashime oil	Methanol	4.25:1 (*n*:*n*)	Whole-cell immobilized	r.t.	>96%	[220]
	*Fusarium heterosporum*	FHL	Soybean oil	Methanol	0.35:9.65 (g:g)	Whole-cell immobilized	30	95%	[221]
	*Fusarium heterosporum*	FHL	Rapeseed oil	Ethanol	0.5:9.65 (g:g)	Whole-cell immobilized	30	94%	[222]
	*Fusarium heterosporum*	FHL	Soybean oil	Methanol	0.35:9.65 (g:g)	Whole-cell immobilized	30	94%	[219]

### 5.2. The Food and Nutraceutical Industries

In the food industry, lipases are used for the synthesis of esters, including short-chain esters, as flavor compounds, and sugar esters as emulsifiers. Other classes of emulsifiers include partial glycerides produced by lipases, such as MAGs and DAGs, and lysolecithin produced by phospholipases. Lipases and phospholipases are used in the dairy industry to accelerate cheese ripening and to improve cheese yield, respectively, and in the baking industry to improve volume, softness and shelf-life of bread and other baked products. Both of these enzymes are important biocatalysts for the synthesis of nutraceuticals, which are bioactive compounds that can provide beneficial effects for human health.

The production of flavors by extraction from the fruit is preferred to chemical synthesis, as the product can then be labelled as “natural”; however, the extraction process entails high cost. At the same time, the use of microbial lipases for the synthesis of flavor compounds represents a good solution, as this is not so expensive and the product obtained can still be labelled as “natural” [89]. As most of these compounds are chiral, flavor synthesis using enantio-selective lipases is preferable to non-selective chemical synthesis. For this reason, the enantio-selectivity of CALB towards secondary alcohols has made this the most widely used lipase in the synthesis of short-chain flavor esters [238]. This is followed by CRL and *Rhizopus* lipases, both in their free and immobilized forms [89]. As the lipase enantio-selectivity depends on efficient immobilization [239], this is strictly required when the flavor compounds being synthesized are chiral.

Recently, lipase-displaying yeast-cell biocatalysts are emerging as an alternative to immobilized lipases. Thus, as shown in Table 5, recombinant CALB displayed on the surface of *S. cerevisiae* cells [240,241] or of *P. pastoris* cells [242,243,244,245] is the form of recombinant lipase that is most widely used in the synthesis of short-chain flavor esters. In this form, the enzyme can produce short-chain esters at high rates, which in some cases were even higher than the rates measured for the commercial Novozym 435 [245]. Interestingly, Jin and coworkers [243] used CALB-displaying *P. pastoris* cells to scale-up the synthesis of 12 kinds of flavor esters in a 5 L batch-stirred reactor. The synthesis yield of 10 of these compounds exceeded 95% after reactions of 4 h, which thus demonstrated the potential commercial applications of this biocatalyst for large-scale production.

Emulsifiers can be endogenously produced or externally added to the processed food. An example of endogenously produced emulsifiers is lysolecithin produced from lecithin hydrolysis after treatment of egg yolk with phospholipases, which is intended for the mayonnaise and sauce industry. Commercial porcine pancreas PLA_2_ (Lecitase^®^ 10L, Novozymes) and *A. niger* PLA_2_ (Maxapal^®^ A2, DSM) have been successfully used for this purpose [75]. For externally added emulsifiers, the most widely used in the food industry are MAGs and DAGs produced by lipase-catalyzed esterification of glycerol with FFAs. The synthesis of MAGs and DAGs is carried out with lipases that show preference for these partial glycerides. In particular, a lipase from *P. camembertii* (Lipase G, Amano) that shows selectivity for MAGs has been commercialized and used for this purpose [246]. Recently, MDL with selectivity for DAGs and MAGs was recombinantly expressed in *P. pastoris*. This enzyme was first used in biodiesel production in association with RML [209], and then it was applied by the same authors to the production of MAGs and DAGs [247] (Table 5). This enzyme was used in the form of a fermentation broth supernatant, and was shown to provide high yields of MAG and DAG production, and to be more efficient than the commercial Lipase G50 when oleic acid was used as the acyl donor. Sugar esters are another class of compounds that are used in food processing for their emulsifying and antifungal properties [73]. Sugar esters can be produced by lipase-catalyzed esterification of a sugar with a FFA, and also in this case, the most commonly used lipase is CALB [248]. In particular, there is evidence that recombinant *P. pastoris* with CALB displayed on its cell surface can act as an efficient whole-cell biocatalyst for sugar monoester production. Indeed, this system has been successfully used for the synthesis of both glucose laurate [249] and fructose laurate [250] (Table 5).

Lipases are extensively used in the dairy industry for selective hydrolysis of milkfat TAGs during cheese ripening. The TAGs of ruminant milkfat are rich in short-chain FAs, and when these are released, they contribute significantly to the flavor of many cheese varieties [77]. The traditional commercial lipases used in the dairy industry are from bovine and porcine pancreas or from pre-gastric tissues of young ruminants (P300, P400 and P600, GetCulture), although many microbial lipases have also been used efficiently, which have included lipases from *A. niger* (Lipase A, Amano), *C. rugosa* (Lipase AY, Amano), *P. roqueforti* (Lipase R, Amano; Lipomod™, Biocatalysts), *P. camemberti* (Lipase G, Amano), *R. javanicus* (Lipase M, Amano), *R. miehei* (Piccnate, Gist-Brocades), and *R. oryzae* (Lipase DF, Amano). A lipase from *R. miehei* that was recombinantly expressed in *A. oryzae* (Palatase^®^ M, Novozymes) has also been commercialized for application in the dairy industry, to improve cheese flavor. Very recently, Peng and coworkers [251] used a novel lipase (Est_p6) from a metagenomic library that they recombinantly expressed in *E. coli* for the hydrolysis of milkfat and to impart a distinctive and desirable flavor to milk products (Table 5). In recent years, there has been increasing interest in phospholipase modifications in the cheese industry, with the discovery that partial hydrolysis of phospholipids results in increased cheese yield. Indeed, a commercial PLA_1_ from *A. oryzae* (YieldMAX^®^ PL, Novozymes), a PLC from *B. cereus*, and a PLD from *Streptomyces chromofuscus* have been used to increase cheese yields [75]. For recombinant phospholipases, Lilbaek and coworkers [252] applied a commercial PLA_1_ from *Fusarium venenatum* expressed in *A. oryzae* (Novozym 46016, Novozymes) in the dairy industry to improve process efficiencies and cheese yields (Table 5).

Lipases and phospholipases have been used in the baking industry since 1990. Their addition to dough allows the hydrolysis of both nonpolar and polar lipids, which results in enhancement of flavor development and *in-situ* generation of emulsifiers. These emulsifiers provide increased stability of the dough to mechanical stress, and thus improve the dough machinability; they also provide increased loaf volume, which results in an improved, more uniform and softer, crumb structure [253]. Currently, various commercial lipases and phospholipases are available for bread baking. These include lipases from *A. niger* (Lipase A, Amano) and *R. oryzae* (Lipase DF, Amano), and PLA_2_s from microbial sources (Lysomax R^®^, Danisco; Maxapal^®^, CakeZyme^®^ and BakeZyme^®^, DSM Food Specialties). Examples of recombinant enzymes that have been commercialized for application in the baking industry include the 1,3-specific lipase from *T. lanuginosus* (Lipopan 50BG^®^, Novozymes) and the lipase/phospholipase from *F. oxysporum* (Lipopan F^®^, Novozymes), both of which are expressed in *A. oryzae*. A new variant called Lipopan^®^ Xtra was recently developed by protein engineering, to complement Lipopan F^®^ [75]. Other applications of recombinant lipases in the baking process are given in Table 5. These include a lipase from *Geotrichum* sp. (LIP 2) [254], whereby dough fermented with *S. cerevisiae* expressing LIP2 provides bread with greater loaf volume and more uniform crumb structure. Similar effects on the rheological properties of bread were observed by Li and coworkers [255], with the addition of dough with a recombinant lipase from RCL in combination with transglutaminase. This combination also provided a product with improved sensorial properties. Recombinant RCL has also been shown to positively affect the thermomechanical properties of dough [256].

Lipases are used to synthesize nutritionally important structured lipids from low-cost oils. These include low-calorie TAGs with short-chain or medium-chain FAs at the *sn*-1 and *sn*-3 positions and long chain FAs at the *sn*-2 position (SLS-TAGs, MLM-TAGs), and TAGs with only medium-chain FAs (MMM-TAGs); TAGs that are rich in ω-3 PUFAs; anti-obesity DAG oil; and human milkfat substitutes.

**Table 5:** Applications of recombinant lipases in food and nutraceutical industry.

Application	Donor Species	Enzyme Name	Host Strain	Lipase Form	Application	Refs.
Flavors	*Candida antarctica*	CALB	*Saccharomyces cerevisiae*	Whole-cell surface-displayed	Ethyl lactate synthesis	[241]
	*Candida antarctica*	CALB	*Saccharomyces cerevisiae*	Whole-cell surface-displayed	Ethyl hexanoate synthesis	[240]
	*Candida antarctica*	CALB	*Pichia pastoris*	Whole-cell surface-displayed	Short chain ester synthesis	[242,243,244]
	*Candida antarctica*	CALB	*Pichia pastoris*	Whole-cell surface-displayed	Short chain ester synthesis	[245]
	*Rhizopus oryzae*	ROL	*Pichia pastoris*	Purified and immobilized	Ethyl butyrate synthesis	[257]
	*Metagenomic library*	LipIAF5-2	*Escherichia coli*	Whole-cell	Short chain ester synthesis	[258]
Emulsifiers	*Penicillium cyclopium*	MDL	*Pichia pastoris*	Crude supernatant	Synthesis of MAGs and DAGs	[247]
	*Candida antarctica*	CALB	*Pichia pastoris*	Whole-cell surface-displayed	Synthesis of fructose laurate	[250]
	*Candida antarctica*	CALB	*Pichia pastoris*	Whole-cell surface-displayed	Synthesis of glucose laurate	[249]
Dairy	*Metagenomic library*	Lipase Est_p6	*Escherichia coli*	Purified	Milk flavor production	[251]
	*Fusarium venenatum*	PLA_1_	*Aspergillus oryzae*	Purified	Mozzarella cheese yield improvement	[252]
Bakery	*Geotrichum* sp.	LIP1 and LIP2	*Saccharomyces cerevisiae*	Whole-cell	Increase in loaf volume and in crumb structure uniformity	[254]
	*Rhizopus chinensis*	RCL	*Pichia pastoris*	Purified	Increase in baking properties of dough	[256]
	*Rhizopus chinensis*	RCL	*Pichia pastoris*	Purified	Increase in loaf volume and in crumb structure uniformity	[255]
Nutraceuticals	*Geobacillus zalihae*	Lipase T1	*Escherichia coli*	Purified	DAG production from palm stearin	[259]
	*Rhizopus oryzae*	ROL	*Pichia pastoris*	Purified	DAG production from soybean oil	[260]
	*Rhizopus oryzae*	ROL	*Pichia pastoris*	Purified and immobilized	HMFS production	[261]
	*Rhizopus oryzae*	ROL	*Pichia pastoris*	Purified and immobilized	HMFS production	[262]
	*Rhizopus oryzae*	ROL	*Pichia pastoris*	Purified and immobilized	Production of MLM-TAGs	[263,264]
	*Geotrichum* sp.	GSL	*Pichia pastoris*	Whole-cell surface-displayed	EPA and DHA enrichment in fish oil	[265]
	*Candida antarctica*		*Pichia pastoris*	Purified and immobilized	Vitamin A ester synthesis	[266]
	*Candida antarctica*	CALA	*Escherichia coli*	Crude supernatant	Removal of *trans* FAs from PHVO	[175]
	*Streptomyces chromofuscus*	PLD	*Pichia pastoris*	Whole-cell surface-displayed	PS production from PC	[267]
	*Streptomyces chromofuscus*	PLD	*Escherichia coli*	Purified	PS production from PC	[268]
	*Streptomyces racemochromogenes*	PLD	*Streptomyces lividans*	Purified	PS and PG production from lecithin	[269]

Lipases have also been used in selective removal of unhealthy *trans*-FAs from partially hydrogenated vegetable oils, and in the synthesis of vitamins and phenolic esters that present higher antioxidant activities and stabilities compared to the corresponding free molecules [89]. Similar to lipases, PLAs and PLBs can be used to incorporate medium-chain and long-chain FAs and PUFAs into phospholipids. The transphosphatidylation reaction catalyzed by PLD is used to produce nonabundant phospholipids with bioactive properties, such as PS, which has an important role in supporting mental functions, and phospholipid-vitamin derivatives that are obtained by introducing water-soluble vitamins as polar head-groups in phospholipids. In this form, vitamins show higher affinities for their biological membranes, where they can exert their antioxidant activities [75].

As shown in Table 5, ROL is the recombinant lipase that has been most widely used in the production of nutraceuticals. For instance, ROL recombinantly expressed in *P. pastoris* and immobilized on macroporous beads has been used to produce low calorie MLM-TAGs, by acidolysis of virgin olive oil with caprylic and capric acids [263,264]. Also, ROL has been successfully applied as a biocatalyst in the production of human milkfat substitutes, by acidolysis of tripalmitine with oleic acid [262], and of lard with FFAs from a fish oil concentrate rich in docosahexaenoic acid [261]. When compared to different commercial enzymes, recombinant ROL has been shown to have levels of incorporation of FFAs similar to those achieved by Novozym 435 and Lipozyme RM IM, and even higher than those obtained with Lipozyme TL [261]. Very recently, Li and coworkers [260] also used recombinant ROL to hydrolyze soybean oil for the production of DAG, a functional oil with benefits for human health. DAG production has also been achieved from hydrolysis of palm stearine using a thermostable lipase (T1 lipase) recombinantly expressed in *E. coli* [259]. Other examples of recombinant lipases that have been used successfully in the synthesis of nutraceuticals include *Geotrichum* sp. lipase (GSL) displayed on the cell surface of *P. pastoris*, which was successfully used to enrich eicosapentaenoic acid and docosahexaenoic acid in fish oil on the basis of selective hydrolysis [265], and a lipase from C*. antarctica* ZJB09193 that was used in high-yield production of vitamin A esters [266]. Also of note, there is the use of engineered CALA variants with acquired *trans*-FA selectivity (see Section 4.2.2) in large-scale removal of *trans*-FAs from partially hydrogenated vegetable oils [175].

Among the recombinant phospholipases, those evaluated for the production of nutraceutical compounds are almost exclusively PLDs from *Streptomyces* spp. For instance, recombinant PLD from *S. chromofuscus* has been used for the conversion of PC and l-serine to PS. This enzyme has been used both in its purified form [268] and displayed on the surface of *P. pastoris* cells [267]; in this latter case, the enzyme remained almost stable after several rounds of use, thus suggesting the potential use of this displaying system for viable industrial production of PS. Similarly, PLD from *Streptomyces racemochromogenes* expressed in the host *Streptomyces lividans* was shown to promote a high rate of conversion of the PC and phosphatidylethanolamine (PE) in soybean lecithin into phosphatidylglycerol and PS, in the presence of glycerol and l-serine, respectively [269]. The conditions to produce this enzyme on a large scale were assessed to define its use for industrial production of phosphatidyl derivatives from lecithin.

### 5.3. Oil Degumming

During storage, vegetable oils form a deposit of so-called gums, which include a variety of compounds, with the main form being phospholipids. These compounds pose many problems for the processing and storage of oils for food and nonfood applications. Indeed, the presence of phospholipids in edible oils can lead to dark-colored oils and the generation of off-flavors [270]. In biodiesel production, a high content of phospholipids in the oil leads to loss of yield, as the FAs enclosed in the phospholipid molecules are not accessible to the lipase for transesterification [271]. Therefore, the removal of nearly all of the phospholipids is essential for the production of high-quality finished oil. For successful oil refining, the phosphorus content should be reduced to less than 10 mg/kg [272]. Hydratable phospholipids can be removed by water degumming [273], whereas the non-hydratable forms are removed by either chemical (*i.e.*, acid or alkali addition) or enzymatic refining processes. Recently, enzymatic oil degumming has been attracting a lot of attention and, to date, enzymatic degumming has already been applied to soybean, sunflower, canola, palm, and several other vegetable oils, with highly satisfactory results. The enzymes most commonly used in degumming of vegetable oils are PLA_1_, PLA_2_ and PLC. All of these phospholipases can reduce the amount of nonhydratable phospholipids, as they cleave them into water-soluble and oil-soluble fragments. The first enzymatic degumming process was developed by the German Company Roehm and Lurgi in the 1990s. The process used, known as the “EnzyMax^®^ process” was based on the use of porcine PLA_2_ (Lecitase^®^ 10L, Novozymes) [274]. More recently, microbial phospholipases suitable for oil degumming have been commercialized both as native enzymes purified from their producing microorganisms, and as recombinant enzymes overexpressed in heterologous hosts. These include two PLA_1_s from *F. oxysporum* (Lecitase^®^ Novo, Novozymes) and *T. lanuginosus*/*F. oxysporum* (Lecitase^®^ Ultra, Novozymes), two PLA_2_s from *T. reesei* (Rohalase^®^ MPL, AB Enzymes) and *A. niger* (Gumzyme™, DSM Food Specialties), a lipid acyl transferase with PLA_2_ activity from *S. violaceoruber* (Lysomax R^®^, Danisco) and a PLC from a *B. anthracis*-like strain (Purifine™, Verenium) [9].

Among the commercial PLAs, Lecitase^®^ Ultra is of particular interest, as a recombinant enzyme that is obtained by fusion of homologous genes that encode *F. oxysporum* phospholipase and *T. lanuginosus* lipase. The chimeric gene was expressed in *A. niger*, and it showed the thermal and pH stability of the *T. lanuginosus* lipase, and the PLA_1_ activity of the *F. oxysporum* phospholipase. When used in oil degumming, this enzyme showed no speficity for any of the phospholipids, and reduced the amount of phosphorous to below the 10 mg/kg, with short reaction times [275]. Lecitase^®^ Ultra has also been immobilized on different solid supports [276]. When applied to soybean oil degumming, immobilized PLA_1_ reduced the phosphorous content to <10 mg/kg, with more than 99% oil recovery.

Purfine™ is also a recombinant enzyme, and it contains a *B. anthracis*-like PLC expressed in *P. pastoris* [9]. Purifine™ catalyzes the splitting off of the phosphate head-group from PC and PE, but has no activity with respect to PI or PA [277]. The DAGs formed during the degumming process using Purifine™ remain in the oil, thus increasing the oil yield; moreover, this enzyme does not produce FFAs that need to be removed later by any refining process, which would inevitably lead to an oil-yield loss. Interestingly, the two recombinant phospholipases Lecitase Ultra^®^ and Purifine™ have been used recently in combination with a lipase (Callera Trans L^®^, Novozymes) both to perform enzymatic degumming and transesterification in a single step, using crude soybean oil as feedstock, and to convert part of the phospholipids into biodiesel [271]. After the combined activity of the different enzymes, more than 95% of the methyl esters were produced, and the phosphorous content was reduced to below 5 mg/kg.

In addition to commercial phospholipases, other recombinant enzymes have been developed and tested for their reduction of the phosphorous content in vegetable oils (Table 6). Very recently, Liu and coworkers [278] expressed a PLA_2_ from *S. violaceoruber* in *P. pastoris*. The recombinant enzyme hydrolyzed PC at a high rate and, when applied in the degumming of rapeseed oil, this PLA_2_ decreased the phosphorous content to 20.74 ± 0.24 mg/kg after a reaction of 6 h, which is still slightly higher than the residual phosphorous content obtained after chemical oil degumming or enzymatic degumming with commercial phospholipases. Nevertheless, the recombinant PLA_2_ from *S. violaceoruber* is very promising for industrial applications, as it has only phospholipase activity, without concomitant lipase activity, which is crucial to avoid oil-yield loss due to TAG hydrolysis. Another successful application of recombinant phospholipases in oil degumming is represented by a PLB from *P. fluorescens* expressed in *P. pastoris* (Pf-PLB-P) [279]. When Pf-PLB-P was used to degum soybean and peanut oils, the phosphorus content decreased to 4.96 and 3.54 mg/kg, respectively, after 5 h of reaction. Similar to the PLA_2_ from *S. violaceoruber*, Pf-PLB-P did not show lipase activity, thus minimizing loss of TAGs during the degumming process.

Recently, the application to oil degumming of phospholipases recombinantly expressed in *E. coli* has also been reported. PLCs from *Clostridium perfringens* were shown to decrease the phosphorous content of rapeseed oil to 5.66 mg/kg after a 1.5 h reaction [280]. The PLC from *Listeria monocytogenes* has been applied to degumming of both soybean and rice bran crude oils, and in both cases, it decreased the phosphorous content to lower than 5 mg/kg [281].

**Table 6:** Vegetable oil degumming by recombinant phospholipases.

Host Strain	Donor Species	Phospholipase Name	Feedstock	Residual P (mg/kg)	Reaction Time (h)	Refs.
*Escherichia coli*	*Clostridium perfringens*	PLC	Rapeseed oil	5.66	1.5	[280]
	*Listeria monocytogenes*	PLC	Soybean rice bran oil	3.70, 4.50	-	[281]
*Pichia pastoris*	*Streptomyces violaceoruber*	PLA_2_	Rapeseed oil	20.74	6	[278]
	*Pseudomonas fluorescens*	PLB	Soybean and peanut oil	4.96, 3.54	5	[279]

### 5.4. The Detergent Industry

Certain enzymes, such as lipase, protease and amylase, have been used as catalysts in detergents since the 1960s to efficiently remove certain stains. In particular, the addition of lipases in detergent formulations is needed to remove fat-containing stains, thus allowing the content of undesirable chemicals to be reduced; moreover, they are biodegradable, leave no harmful residues, have no negative impact on sewage treatment processes, and pose no risk to aquatic life. Lipases used in detergent formulations are chosen on the basis of their broad substrate specificity, and their resistance to the harsh washing conditions of alkaline pHs and high temperatures, to oxidizing and chelating agents, and to damage induced by surfactants and other enzymes, such as proteases, which are found in many detergent formulations [131]. In recent years, the trend toward low washing temperatures has made the removal of fatty stains more difficult. The use of cold-active lipases in the formulation of detergents has solved this problem, with the additional advantages of a reduction in energy consumption and wear and tear of the textiles [282].

The first lipase introduced into detergent formulations was commercialized in 1988 by the company Novo Nordisk. This enzyme is known as Lipolase^®^, and it was naturally produced by a selected strain of *T. lanuginosus*, although at concentrations too low for industrial applications. So, to enhance the production yield, the gene that encodes this lipase was cloned and inserted into the host *A. oryzae*. This heterologous system allowed its expression to reach commercially significant levels of production. In the 1990s, other recombinant lipases were introduced onto the market for detergent applications. In particular, two lipases from *P. mendocina* and *P. alcaligenes*, which are known as Lumafast^®^ and Lipomax^®^, respectively, were commercialized by Genencor (now Du Pont), while a lipase from *P. glumae* was commercialized by Unilever. All these three enzymes were recombinantly expressed in *B. subtilis* [91].

In the subsequent years, these enzymes have been subjected to protein engineering to improve their stabilities and catalytic properties under washing conditions [283]. For instance, different variants of Lipolase^®^ were obtained by SDM approaches. In particular, this enzyme was engineered to improve its binding to fatty stains. To achieve this goal, the negatively charged aspartate residue in the lipid contact zone was exchanged for an uncharged leucine residue. In this way, a new enzyme was obtained, then called Lipolase^®^ Ultra, with improved washing performance at low temperature. Moreover, the T231R and N233R mutations generated new Lipolase^®^ variants, known as Lipex^®^, which showed better performances in the first wash at temperatures as low as 20 °C and showed beneficial synergistic results when combined with bleach catalysts. Another engineering variant of Lipolase^®^ that shows better first washing performance is LipoPrime^®^, which carries the mutation S216W. For the *P. alcaligenes* lipase, Lipomax^®^ is an engineered variant of the WT enzyme in which the substitution of the methionine at position 21 with leucine resulted in increased washing performance of this lipase. This enzyme was also engineered for improved stability with anionic surfactants, which are responsible for the inactivation of the enzyme in liquid detergents. To block the entrance of the anionic surfactant into the enzyme, and to prevent denaturation of the enzyme, the positively charged amino acids on the protein surface were substituted with negatively charged amino acids, and a new more stable variant was obtained. Also, a lipase from *P. glumae* was engineered to improve its resistance to proteases. To achieve this goal, mutations were introduced in the cleavage sites, to introduce amino-acid residues that are not preferred by proteases. Moreover, the M254I mutation was also introduced to improve the stability of this enzyme in the presence of oxidizing agents.

Very recently, Kranen and coworkers [284] applied a whole-cell biocatalyst in the real-life laundry process for the first time. A lipase from *B. cepacia* and its chaperone, the so-called lipase-specific foldase (Lif) that is responsible for correct folding of the lipase, were co-expressed on the surface of *E. coli* via autodisplay. The whole-cell biocatalyst and the outer-membrane preparations were used in a standardized laundry tests that imitated conventional machine washing. Both of these preparations appeared to be stable enough to endure the standard European laundry test, while they showed the same lipolytic activity as the purified lipase from *B. cepacia* and the commercial Lipex^®^. These results clearly show that autodisplay represents a more convenient alternative to obtain a functional biocatalyst without the need for prior laborious purification steps, and that successful removal of fat or grease spots can be achieved by simply working with a cell-free preparation containing *E. coli* outer membrane preparations that contain the active lipase.

## 6. Conclusions

The use of lipases and phospholipases as biocatalysts provides an attractive and environmentally friendly alternative to the conventional chemical approach. However, despite lipase-catalyzed and phospholipase-catalyzed processes having great commercial significance, the use of these enzymes is not extensive, because of the low reproducibility, low yields and the not-always-optimal performance of these biocatalysts in their native form. The examples reported in this review show that recombinant DNA technology and protein engineering are growing rapidly and might represent a suitable way to overcome these drawbacks and to produce robust enzyme catalysts at high yields.

With the growing knowledge of the machinery for the regulation of gene expression, functional improvement of the expression systems is currently in progress, and new overproducing strains are continuously emerging. Such increased yields in the production of recombinant biocatalysts are economically advantageous, not only for increased volumetric productivity, but also because of reduced downstream purifications costs, and with the resulting crude enzyme being much purer. Interestingly, in the last few years, whole-cell systems with the biocatalyst on the cell surface have emerged as possible solutions to overcome some of the technical and economical limitations associated with enzyme purification and immobilization.

Furthermore, an increased understanding of the primary and three-dimensional structures of the enzymes, the growing number of gene sequences deposited in public databases and in environmental DNA libraries, together with the development of new tools for *in-silico* construction of protein mutants and the prediction of their activities, are all directing protein engineering towards more focused approaches that combine rational design and directed evolution. This will significantly reduce the screening efforts needed, and thus facilitate the identification of novel and useful enzymatic variants.

Altogether, for the near future, these improvements will yield promising outcomes for the widespread industrial applications of lipases and phospholipases as biocatalysts.

## Abbreviations

Aox, alcohol oxidase; *a*_w_, water activity; C2-PLD, N-terminal C2 Ca^2+^-binding domain-PLD; CALA, *Candida antarctica* lipase A; CALB, *Candida antarctica* lipase B; CASTing, combinatorial active site saturation test; cPLA_2_, cytosolic, Ca^2+^-dependent PLA_2_; CRL, *Candida rugosa* lipase; DAD1, Defective in Anther Dehiscence 1; DAG, diacylglycerol; DHA, docosahexaenoic acid; DMSO, dimethyl sulfoxide; EPA, eicosapentaenoic acid; epPCR, error-prone polymerase chain reaction; FA, fatty acid; FAAE, fatty acid alkyl ester; FFA, free fatty acid; FHL, *Fusarium heterosporum* lipase; GRAS, Generally Recognized As Safe; GSL, *Geotrichum* sp. lipase; HMFS, human milkfat substitutes; iPLA_2_, cytosolic, Ca^2+^-independent PLA_2_; IPTG, isopropyl β-D-1-thiogalactopyranoside; LED, Lipase Engineering Database; Lif, lipase-specific foldase; L-PLA_2_, lysosomal PLA_2_; MAG, monoacylglycerol; MDL, *Penicilium cyclopium* MAG- and DAG-specific lipase; MLM-TAG, TAG with medium-chain FAs at the *sn*-1 and *sn*-3 positions and long chain FA at the *sn*-2 position; MMM-TAG, TAG with only medium-chain FAs; mPA-PLA_1_, membrane-associated phosphatidic-acid-specific PLA_1_; NPC, non-specific PLC; OMPLA, outer-membrane PLA; PA, phosphatidic acid; PAF-AH, platelet-activating-factor acylhydrolase; PA-PLA_1_, phosphatidic-acid-specific PLA_1_; PC, phosphatidylcholine; PC-PLC, phosphatidylcholine-specific PLC; PE, phosphatidylethanolamine; PG, phosphatidylglycerol; PHVO, partially hydrogenated vegetable oil; PI, phosphatidylinositol; PI-PLC, phosphatidylinositol-specific PLC; PLA_1_, phospholipase A_1_; PLA_2_, phospholipase A_2_; PLB, pPhospholipase B; PLC, phospholipase C; PLD, phospholipase D; PLRP, pancreatic lipase-related protein; PS, phosphatidylserine; PS-PLA_1_, phosphatidylserine-specific PLA_1_; PUFA, polyunsaturated fatty acid; PXPH-PLD, N-terminal phox homology (PX) and pleckstrin homology (PH) domains-PLD; RCL, *Rhizopus chinensis* lipase; RML, *Rhizomucor miehei* lipase; ROL, *Rhizopus oryzae* lipase; SDM, site-directed mutagenesis; SLS-TAG, TAG with short-chain FAs at the *sn*-1 and *sn*-3 positions and long chain FA at the *sn*-2 position; sPLA_2_, secretory PLA_2_; SSM, Site saturation mutagenesis; TAG, triacylglycerol; TLL, *Thermomyces lanuginosus* lipase; TLPLB, *Thermotoga lettingae* PLB; WT, wild-type; YlLip2, *Yarrowia lipolytica* lipase 2.
